# Gut microbiota-gonadal axis: the impact of gut microbiota on reproductive functions

**DOI:** 10.3389/fimmu.2024.1346035

**Published:** 2024-02-28

**Authors:** Victory J. Ashonibare, Bolaji A. Akorede, Precious J. Ashonibare, Tunmise M. Akhigbe, Roland Eghoghosoa Akhigbe

**Affiliations:** ^1^ Department of Infection Biology, Leibniz Institute for Natural Product Research and Infection Biology, Jena, Germany; ^2^ Institute of Microbiology, Friedrich Schiller University, Jena, Germany; ^3^ Reproductive Biology and Toxicology Research Laboratory, Oasis of Grace Hospital, Osogbo, Nigeria; ^4^ Department of Biomedical Sciences, University of Wyoming, Laramie, WY, United States; ^5^ Department of Physiology, Ladoke Akintola University of Technology, Ogbomoso, Oyo State, Nigeria; ^6^ Breeding and Genetic Unit, Department of Agronomy, Osun State University, Ejigbo, Osun State, Nigeria

**Keywords:** gut microbiota, infertility, inflammation, insulin resistance, microbiome, oxidative stress

## Abstract

The influence of gut microbiota on physiological processes is rapidly gaining attention globally. Despite being under-studied, there are available data demonstrating a gut microbiota-gonadal cross-talk, and the importance of this axis in reproduction. This study reviews the impacts of gut microbiota on reproduction. In addition, the possible mechanisms by which gut microbiota modulates male and female reproduction are presented. Databases, including Embase, Google scholar, Pubmed/Medline, Scopus, and Web of Science, were explored using relevant key words. Findings showed that gut microbiota promotes gonadal functions by modulating the circulating levels of steroid sex hormones, insulin sensitivity, immune system, and gonadal microbiota. Gut microbiota also alters ROS generation and the activation of cytokine accumulation. In conclusion, available data demonstrate the existence of a gut microbiota-gonadal axis, and role of this axis on gonadal functions. However, majority of the data were compelling evidences from animal studies with a great dearth of human data. Therefore, human studies validating the reports of experimental studies using animal models are important.

## Introduction

### Sources of gut microbiota

The human body contains countless microorganisms, which makes the body a planet filled with ecosystems. Most of these reside in the gut, while others reside in the mouth (1), skin (2), vagina (3), and penis (4). The microbiome of individuals is unique to each person, just like the fingerprint and genome. From where do they originate? The human body serves as the largest reservoir of gut microflora. They transmit the microbes from another reservoir to reservoir. In addition, food, water, the environment, and animal also carry microorganisms that make up the human gut (5).

The birth of a child is the very first form of acquisition and transmission of gut microflora. The source of the initial microorganism depends on the mode of delivery. As a child passes through the birth canal of a mother, it comes in contact with its primary microflora from the mother through the vaginal, via the faeco-oral or vaginal-oral route, while those born through caesarean section acquire theirs through the skin. It further encounters other bacteria and organisms through skin- to-skin contact, and breastfeeding. The exposure to these microorganisms is known as seeding. Thus, unlike the genome, microbiome composition originates from the biological mother. As the child is exposed to the world, the composition of its microbiome is influenced by factors such as the birth and growth environment, nature, nutrition, other members of the family, pets among others (6). Seeding is pertinent to the biological development of a child. The microbes colonize the gastrointestinal tract (GIT) and multiply rapidly, this ensures longevity of the microorganisms. Transmission of gut bacteria to the new born continues upon birth as the baby comes in contact with other humans, especially members of the family. Transmission also occurs from pets and the environment in which the child lives in its early life (5). The takeover by obligate anaerobes is determined by transmission ability among human population, that is, the ability to exit a host, enter and colonize another (7). At birth and through the first three years of life, the GIT is first dominated by facultative anaerobic bacteria, which are later replaced by obligate anaerobes as the child transitions to eating solid food (8, 9).

Some animals share similar microbiome with humans; *Roseburia*, *Faecalibacterium*, *Bacteroides*, *Prevotella* and *Ruminoccocus* are commensal bacteria found in humans, dogs, and cats (10, 11), while intestinal infections caused by *Salmonella enterica* subsp. *enterica* serovar Enteritidis, enteropathogenic *E. coli, Campylobacter jejuni*, and *C. difficile* are transmitted between animals and humans (12–14). The interaction between humans and animals has also contributed to the incessant spread of antibiotic resistance. Therefore, there is a possibility that commensal bacteria are transmitted from animals to humans and vice versa. Interaction with pets and farm animals is thus a source of acquiring gut microbiota.

Foods contain microorganisms that could make up the gut microflora (15). Breast milk supplies a baby about 8 million intestinal bacteria on a daily basis (5). It has been established that humans consume about 10^6^ to 10^9^ microorganisms daily from food. Although not all these survive the digestion process and those that do survive do not colonize the gut for a long term, gut microbiota acquired through food are obtained through horizontal gene transfer. Food serves as a source of external bacteria species and as genes for commensal gut microbes to acquire. Probiotics, prebiotics, and synbiotics also influence the gut microbiota.

**Figure 1:**
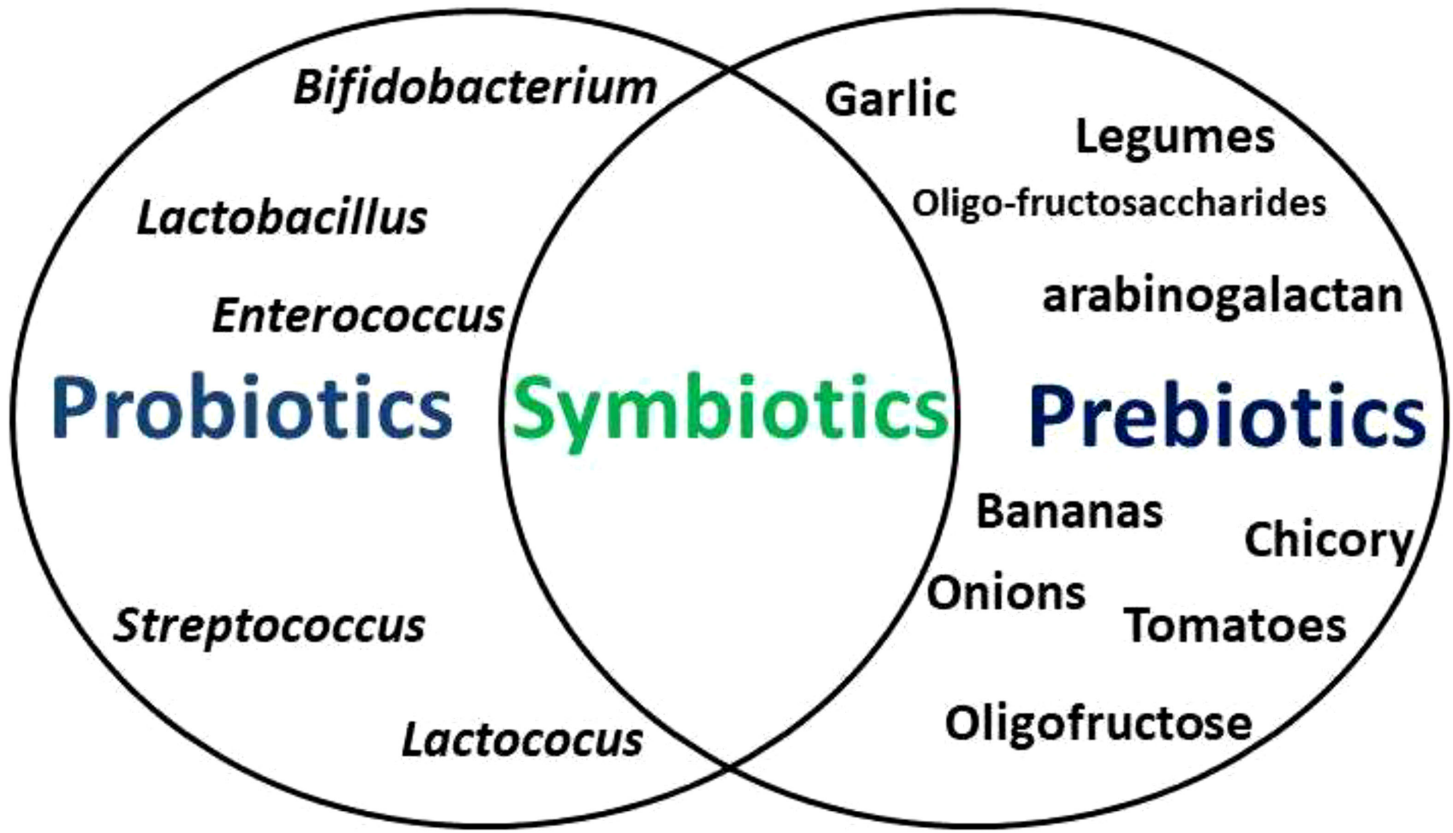
The association between probiotics, prebiotics, and symbiotics. Probiotics are viable bacteria and yeasts that confer health benefits when consumed in the appropriate proportions, usually as food supplements or with some foods, while prebiotics are fibre-rich foods that support the growth of human microflora. When both are taken together, this becomes symbiotic.

Probiotics are viable bacteria and yeasts (predominantly *Bifidobacterium* and *Lactobacillus, Lactococus*, *Streptococcus*, *Enterococcus* species) (16) that confer health benefits when consumed in the right quantity, usually as food supplements or with some foods (17). Prebiotics are fibre-rich foods that support the growth of human microflora (18). When both are taken together, this becomes symbiotic (**Figure 1**). Of the numerous benefits of probiotics, they mainly are involved in the development of normal flora of the gut in order to ensure a balance between invaders and bacteria responsible for normal functioning of the organism (19, 20). Probiotics restore the natural microbiome of the gut after drug therapy (21, 22). Studies have shown that prebiotics (artichokes, asparagus, bananas, berries, chicory, garlic, green vegetables, legumes, onions, tomatoes, as well as barley, cereals, linseed, oats, vegetables, and wheat Fruit) modify the growth of gut bacteria. They selectively foster the growth of microorganisms in hosts gut and modify the gut environment such that normal flora can effortlessly grow and reproduce, but unconducive for pathogens of the gut (23, 24).

The environment is another reservoir of microorganisms; indoor airborne microbes circulate through ventilation systems, while outdoor organisms could be carried by humans to become inborne. Bacteria reside on surfaces within and outside the home environment, many of which are skin-resident. However, intestinal bacteria belonging to the families of *Bacteroidaceae*, *Prevotellaceae*, *Ruminococcaceae*, and *Lachnospiraceae* have been isolated from bathroom and toilet surfaces (25, 26), and could be transmitted into human gut via poor hygiene. Water harbours a lot of intestinal pathogens, which are linked to gastrointestinal diseases. When improperly treated water is consumed, there is a risk of consuming bacteria pathogens such as *Shigella sonnei*, *Shigella flexneri*, and *V. cholerae.* The means of transmission is not fully understood, but *Blautia spp and E. coli* have been isolated from water and linked to be of human origin (27).

### Disruption of gut microbiota

The microbial composition gets perturbed by very many factors, which can alter or destroy the function and makeup of the microbiome. The gut microbiome is in a constant state of change through life; its role in both health and disease are been studied. Studies have established the link between gut microflora and human metabolism, nutrition, physiology, and immune function. The exact contribution to disease progression is not clear, however, a disruption of these commensal microbes is an environmental factor that impacts on hosts metabolism and plays a role in diseases such as diabetes, obesity, and atopy- and gut-related Irritable bowel syndrome IBS, and Inflammatory Bowel Disease, IBD (28). The microbes in the child reach a steady state around age 2 or 3; these ecosystems are however altered by external factors to form the composition which dominates through the entire life of the child (29, 30). If the microbiota would return to its previous state after a disruption is determined by the extent of disruption, exposure to other microbes, and the composition of microbiota.

Food is one of the factors that influences the abundance and diversity of the gut microorganisms. Certain foods have been linked with the general state of health by affecting the microflora of the intestinal tract. According to a study by McIntosh et al. (31), low fermentable oligosaccharides, disaccharides, monosaccharides, and polyols (FODMAP) diet such as dairy, fruits, vegetables, proteins, nuts and seeds, grains increased Actinobacteria in the gut, while high FODMAP diet reduced bacteria that in turn produced gas. Uchida et al. (32) demonstrated that cheese increased the abundance of *Bifidobacterium* and Foligné et al. (33) showed that cheese also decreased *Bacteroides* and *Clostridia*; some of these strains of bacteria are culprits in gut infections. Food additives, high intensity sweeteners, polyphenols from tea, coffee, berries, and some vegetables have also been proven to influence the gut microbial diversity (34–40).

Also, drugs are important modulators of the gut microbial composition. Many researchers have studied how commonly used drugs alter the composition, functions, and abundance gut microbiota (41, 42). Weersma and others (43) reported on how 19 groups of commonly administered drugs modulate different gut microflora among Belgium Flemish people. ACE inhibitors, beta-blockers, laxatives, lipid-lowering statins, metformin, proton pump inhibitors (PPI), and selective serotonin reuptake inhibitor antidepressant have been reported to modulate gut microbiota (43–45). A study in the Netherlands reported a decrease in the diversity of gut microbes with the use of PPIs (41), which agrees with the reports by Imhann et al. (46) who opined that PPIs altered the bacterial population among some populations; in the report, some population increased while others decreased. A similar study reported a decrease in microbial diversity of gut microflora from faecal samples obtained from the cohorts (47). In general, PPIs alter commensal organisms of the intestine (*Enterobacteriaceae*, *Enterococcaceae*, and *Lactobacillaceae*) decrease *Ruminococcaceae* and *Bifidobacteriaceae*, but increase bacteria resident in the oral cavity (*Rothia dentocariosa* and *Rothia mucilaginosa*, the genus *Actinomyces* and the family *Micrococcaceae*) (46). Another drug with a wide range of application, which has a modulatory effect on gut microbiome is Metformin. It is used to control blood glucose levels and prevent complications such as renal injury, blindness, and sexual/erectile dysfunction in diabetic patients. Although its mode of action is not fully understood, it has been reported to cause a change in gut bacteria. It was reported that the use of metformin among a group of people resulted in a change in over 80 species of bacteria when compared to the control group. The use of metformin caused a significant increase in *Escherichia coli* and reduced *Intestinibacter.* In addition, the study reports one- third of the total population to which metformin was administered suffered gastrointestinal disorders such as like diarrhoea, bloating and nausea, which were caused by an increase in *Escherichia coli* population (48, 49). In addition to metformin and PPIs, other commonly used drugs such as laxatives, statins, antidepressants and opioids have been reported to influence gut microbiome (41, 44, 46). An increase in *Bacteroides* species has been reported in patients on laxatives, which is similar to the findings in mice that were administered polyethylene glycol (PEG) (50–52). Similarly in a study, the authors administered broad spectrum antibiotics consisting of neomycin, vancomycin, and metronidazole to 11 human cohort suffering bacteria gastrointestinal infection for 5 days, with the aim to measure the effect of these antimicrobials on gut microbiota (53). The study showed a non-negligible change in the composition and diversity of the microbiome, with the highest alteration occurring one month after antibiotic intervention. Specifically, *Enterobacteriaceae* remained dominant till the 7th day post antibiotic therapy. By the 30th day, *Lachnospiraceae*, *Enterobacteriaceae*, and *Ruminococcaceae* were greatly reduced but finally returned to their previous state by day 90 post-antibiotics (53). The entry of an invading microorganism, which successfully colonizes the gut and competes with the normal flora for space and nutrient may cause a depletion of the resident flora and outnumber same.

### Immunomodulatory effects of gut microbiota

Since there are several factors that disrupt the human microbiota, it is therefore almost impossible to define a healthy microbiota. This large variability could cause commensal and/or mutualistic microorganisms to turn pathogenic. The opportunistic invasion could result in infection and inflammation. A healthy microbiota is one that returns to its previous state after recovering from a disruption (54). The immune system maintains a constant symbiotic relationship with microorganisms to maintain a state of balance. These microbial populations control the host’s physiological and metabolic functions, they are involved in the maturation of intestinal immune cells (55, 56) and maintaining homeostasis, as well as exert strong immunomodulatory effects in response to invasions (57). Although the exact mechanisms have not been fully elucidated, studies have demonstrated that the interaction between gut microbiota and the host immune system undeniably impacts inflammation and glucose tolerance. Gut microbiota plays an important role in the maturation of CD4 + TH cells, which is crucial for host defense and the development of autoimmune disease by producing pro-inflammatory cytokines (58). Certain commensal bacteria of the guts are responsible for the induction of Treg cells (58). In addition, immunoglobulins and innate lymphoid cells (ILCs) are also dependent on this microbial community for development (58). Gut microbiota shapes the transcriptional landscape of the hepatic endothelium, thus modulating hepatic endothelial sphingosine metabolism and the sphingosine-1-phosphate pathway (59).

A study by Zhao et al. (60) showed that *Akkermansia muciniphila* supplementation repressed metabolic inflammation in mice fed a chow diet. This study demonstrated that *A. muciniphila*, a gut bacterium, regulates host immune response by inhibiting inflammatory pathways, ER stress, and lipogenesis in insulin-responsive tissues, leading to improved insulin action and glucose tolerance (60). *A. muciniphila* protects the gut from invasion and infections (61). The study in addition to this reported an increase in α-tocopherol, β-sitosterol (60). Another study with a gnotobiotic mouse model carried out by Desai et al. (62) that aimed at studying the relationship between dietary fiber deprivation on gut microbiota and the mucus defense effect, showed that a malfunction of gut microbiota results in inflammation and increased susceptibility to invasion, which arises from the degradation of the colonic mucus barrier. The mucus barrier is made up of antimicrobial peptides and immunoglobulins, which a potential microorganism must successfully bypass to cause an infection (63). Sonnenburg et al. (64) opined that there is a connection between diet and the mucus barrier. Authors have as well reported depletion of the colonic mucus barrier as a response to reduced dietary fiber (65–67). Other studies suggested these diets support the growth of normal flora of the gut. A disruption in the population and physiology of gut microflora (gut dysbiosis) is implicated in the pathogenesis of diseases, including host susceptibility to pathogens, inflammatory bowel disease (IBD), and colon cancer (68, 69). Successful treatment of gut dysbiosis negatively modulates inflammasomes and represses unsolicited immune system activation (54). Gut microbiota ensures a balance in mucus secretion and production. As reported, an imbalance of mucus production leads to inflammation of the intestine (70) and supports the entry and invasion of commensal bacteria in the inner mucus layer in murine models of colitis and ulcerative colitis patients (71).

#### Gut-microbiota modulation of innate immunity

In a balanced system, phagocytes are sequestered within the lamina propria. This is necessary to ensure that the immune system maintains a state of unresponsiveness to commensal bacteria. The phagocytes are not activated as long as the epithelial barrier is not compromised. However, the immune system becomes activated through a cascade of processes once an invader/pathogen is detected. *S. Typhimurium* and *Pseudomonas aeruginosa* promote *caspase1*/Interleukin-1 converting enzyme (ICE) by inducing pro-inflammatory IL-1β (58, 72). When active, *caspase 1* cleaves inactive inflammatory cytokines IL‐1β and ‐18 and converts them to their active forms. The cytokines thus activate other immune cells to attack and ward off the invading pathogens (58, 72). Growing evidence has shown that gut microbiota regulates T lymphocytes (73). Some studies have suggested as well that the development of B-cells takes place in the intestinal mucus, and it is controlled by signals from commensal microorganisms, resident in the gut (74). Kamada et al. (58) also posited that the gut microorganisms positively modulate innate immunity by stimulating ILCs to produce IL-22. This is in agreement with other authors that documented that the production of IL-22 likely depends on commensal gut bacteria or their metabolites, as germ-free mice lacked the ability to produce IL-22 (75). Mice lacking the IL-22 production cells were more susceptible to *C. rodentium* infection than their counterparts. This suggests that IL-22 production, which is gut microbiota-dependent, is crucial for protection against pathogen invasion. Summarily, gut microorganisms might modulate host defense by activating the production of IL-22 through ILC stimulation. Gut microbiota has also been reported to suppress neutrophil extracellular traps (NET)ing neutrophil hyperactivity in mesenteric ischaemia/reperfusion injury, while ensuring immunovigilance by enhancing neutrophil accumulation (76).

#### Gut-microbiota modulation of adaptive immunity

As previously mentioned, some intestinal microbiota regulates the production of T lymphocytes, which play important roles in the pathogenesis of some diseases (58, 77–82). TH17 cell differentiation is induced by the colonization by segmented filamentous bacteria (SFB), which confers protection against *C. rodentium* invasion (78). There is growing evidence that TH17 cells are essential in regulating immune responses in the intestine and that they protect against some pathogens. SFB are commensal organisms that colonize the epithelia of the host ileum; they are attached to the surface of the absorptive gut epithelium but do not induce inflammatory responses (83). Although the presence of SFB in humans is still debatable, some studies have reported the isolation of representative members such as *Eubacterium, Prevotella*, *Roseburia, Escherichia*, and *Klebsiella Clostridia* spp from human intestinal mucosa (2, 84, 85).

Furthermore, the hyper reaction of immune cells to invading pathogens could result in damage to the host intestinal mucosa. Treg cells regulate the intensity of immune responses in order to prevent host damage (81). As previously stated, the production of Treg cells is gut microbiota-induced. Thus, gut microbiota regulates the host’s immune protection. Studies demonstrate that *B. fragilis* plays a crucial role in promoting IL-10-producing Treg cells, which fight against invasion of the host by *Helicobacter hepaticus* (81), *Bifidobacterium infantis* (86), and reduce the severity of *S. Typhimurium* infection (87). Gut bacteria have as well been reported to play a role in the production of IgA and CD4+ T cells. These immune cells target specific antigens (88–90). The exact role and mechanisms by which gut microflora regulate adaptive immune responses is still under investigation, but based on evidence from different studies, commensal organisms of the intestinal mucosa play important roles in activating various immune cells that serve as barriers for invaders and prevent epithelial invasion and disruption; they as well contribute to clearing off pathogens via opsonization. It is at least safe to say gut microbiota release microbial molecules that enhance host defense responses (58, 91).

Putting together, commensal microbes protect the host from pathogen invasion, prevent infections, limit the severity of infection, and are involved in pathogen clearance upon infection of the gut. In addition, they play important roles in the upregulation and downregulation of immune cells and are crucial to maintaining homeostasis.

## Gut microbiota and reproductive functions

### Gut microbiota and female reproduction

**Figure 2:**
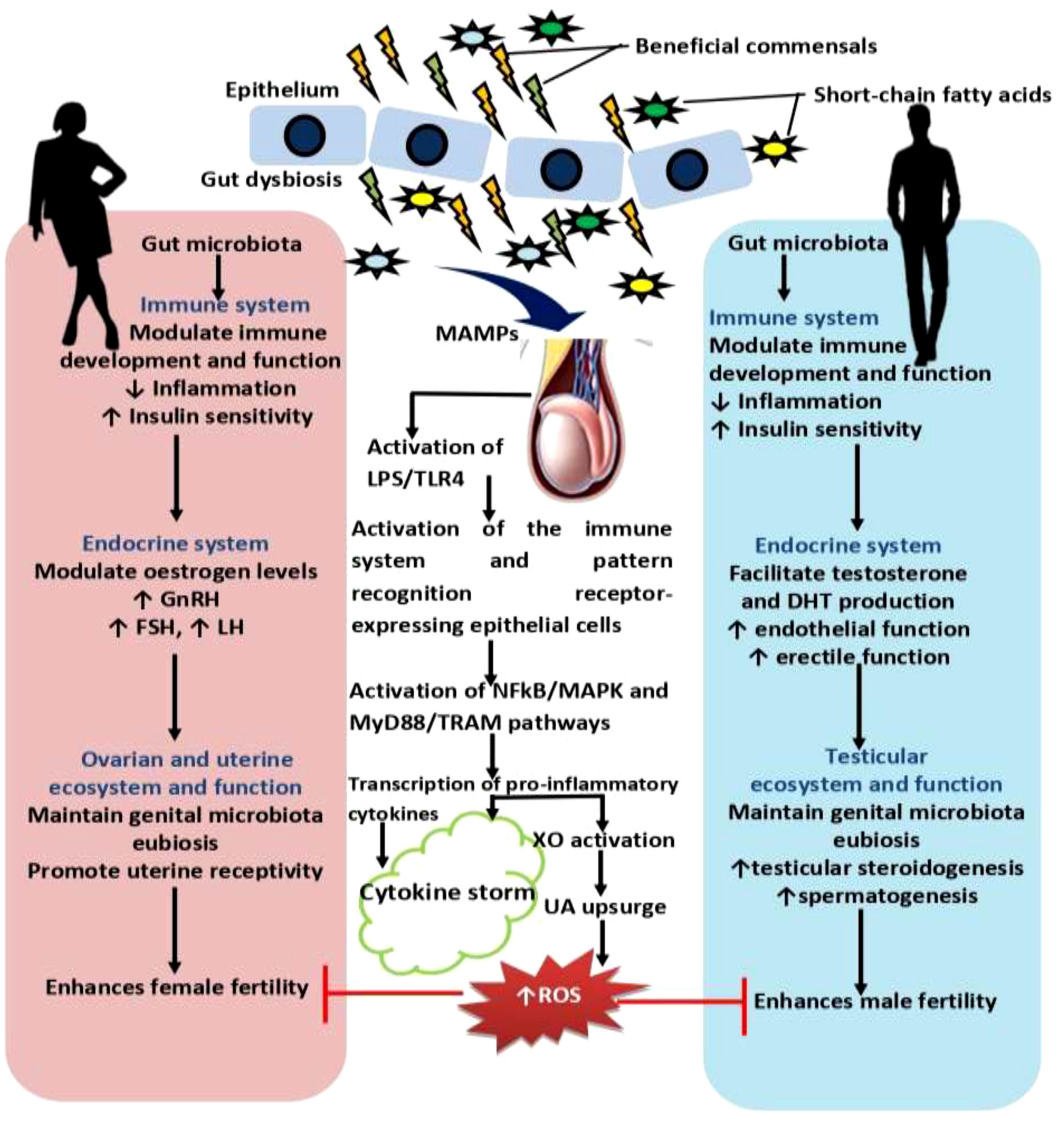
Effect of gut microbiota and dysbiosis on male and female reproductive function. Gut microbiota promotes innate and adaptive immune development and function, and control inflammatory response, which in turn enhances insulin sensitivity. Improved insulin sensitivity and the maintenance of balance redox state facilitates the hypothalamic-pituitary-gonadal axis, culminating in optimal levels of gonadotropin releasing hormone, GnRH, follicle-stimulating hormone, FSH, and lutenising hormone, LH that results in optimal ovarian steroidogenesis (with optimal oestrogen production), testicular steroidogenesis (with optimal testosterone and dihydrotestosterone, DHT, production), and spermatogenesis. These events promote reproductive functions. However, gut dysbiosis promotes the translocation of microbial-associated molecular patterns, MAMPs, such as lipoprotein acids, lipoproteins, peptidoglycans, and lipopolysaccharide, LPS from the gut through the circulation via the hepatic portal vein or lymphatic system into the gonads to induce hyper-immunological response, chronic inflammation, and gonadal damage by activating the innate immune cells and pattern recognition receptors-expressing epithelial cells through LPS/toll like receptor 4 (TLR4), nuclear factor kappa-B (NFkB)/mitogen-activated protein kinases (MAPK), and MyD88 and TRAM-dependent signaling pathways. Activation of these pro-inflammatory processes stimulate xanthine oxidase, leading to increased uric acid generation and oxidative stress that causes ovarian and testicular injury and impairs fertility.

The gut microbiota plays an essential role in several physiological processes, including reproductive function. The influence of gut microbiota on female reproduction is an area of growing interest and research (92). Valeri and Endres (93) discovered that the gut microbiota has both direct and indirect effects on female reproductive health. Gut microbiota influences reproductive function, especially female reproductive functions, through various mechanisms, including hormone regulation, immune system modulation, nutrient metabolism, inflammatory pathways, and genital ecosystem pathway (94) (**Figure 2**). The metabolism and regulation of circulating estrogen hormones are carried out by the enzyme GUSB, which is one of the many enzymes involved in host metabolism that are encoded by the gut microbiota (95). There is a dearth of data linking gut microbiota and female reproduction and many aspects of the gut microbiota’s influence on female reproduction are yet to be fully elucidated, nonetheless, understanding and optimizing the gut microbiota’s role in reproductive health hold promises for improving women’s reproductive outcomes and overall well-being.

The gut microbiota is involved in the metabolism and regulation of hormones that are essential for female reproductive function (96) and dysbiosis in the gut microbiota have been associated with altered hormone levels, which can disrupt normal female reproductive processes (97). For example, it has been shown to influence the metabolism of *estrogen*, a key hormone involved in the menstrual cycle and fertility. Indeed, as earlier stated, the gut microbiome encodes different enzymes involved in host metabolism, certain bacterial species in the gut produce enzymes that can modify estrogen affecting their bioavailability and activity in the body. The circulating estrogen is metabolized and modulated by the enzyme GUSB, which secretes β-glucuronidase, an enzyme that deconjugates estrogen and allows it to bind to estrogen receptors (92). Additionally, gut microbes can produce or modify other hormones, such as progesterone and follicle-stimulating hormone (FSH) (98), which are vital for the menstrual cycle, follicle development, ovulation, and physiological downstream effects (99, 100). Thus, modifications to the microbial community that codes for the GUSB enzyme, referred to as the estrobolome (101), influence enterohepatic circulation of these hormones, which in turn affects endogenous estrogen metabolism and ultimately affects hormonal balance and fertility (94). This microbial influence on estrogen metabolism may affect menstrual regularity, ovulation, and overall reproductive health.

Women with endometriosis have been shown to have a diminished *Lactobacillus* spp. dominance, an altered *Firmicutes*: *Bacteroidetes* ratio, and an abundance of vaginosis-related bacteria with other opportunistic pathogens (102, 103). This may be accompanied by an upregulation of ovarian estrogen secretion via neuro-active metabolites that excite GnRH neurons, which in turn worsens hormonal homeostasis (103). Also, PCOS patients show an abnormal *Escherichia*: *Shigella* ratio and an excess of *Bacteroides* compared to healthy women (104, 105), which is associated with insulin resistance that is characterized by an increased *Firmicutes*: *Bacteroidetes* ratio as seen in endometriosis. Moreover, gut microbiota exerts a role in the pathogenesis of thyroid autoimmune disease, an endocrinopathy that is usually associated with infertility (106–108).

Since a healthy gut microbiota and the immune system have a significant association, the gut microbiota and female infertility appear to be intimately related (109). The gut microbiota plays a crucial role in regulating the immune system. Gut microbial dysbiosis can trigger immune system dysfunction and chronic low-grade inflammation. This inflammation can affect the female reproductive organs, leading to infertility-related disorders like endometriosis (102, 103, 110), polycystic ovary syndrome (PCOS), insulin resistance (IR) (104, 110–115), and obesity (104, 113) characterized by an altered immune profile and pro-inflammatory status, known to adversely affect fertility (116, 117). Reduced gut microbiota diversification and particular microbial imbalances in the gut and reproductive tract are the defining characteristics of these disorders, which result in immunological dysfunction, impaired immunosurveillance, and disrupted immune cell profiles. Additionally, a connection between premature ovarian insufficiency (POI) and the gut microbiota has been proposed (118, 119).

Furthermore, the gut microbiota helps educate and shape the immune system by modulating the development and function of immune cells that are involved in reproductive processes (120). Imbalances in the gut microbiota during critical developmental periods may disrupt immune programming, potentially impacting fertility and pregnancy outcomes (95).

The gut microbiota modulates female reproductive function via its crucial role in the digestion and absorption of nutrients from the diet. It can produce enzymes and metabolites that influence the breakdown and utilization of various nutrients, including vitamins, minerals, and macronutrients (121). Optimal nutrient metabolism is essential for reproductive health as it provides the necessary building blocks for hormone synthesis, energy production, and overall cellular function. Imbalances in the gut microbiota can affect nutrient absorption and utilization, potentially leading to deficiencies or excesses in key nutrients that are essential for reproductive processes (122). Inadequate absorption of certain vitamins or minerals may impair ovulation, embryo development, and implantation (123, 124).

Chronic low-grade inflammation, often associated with dysbiosis, can have detrimental effects on female reproductive health (125, 126). Inflammatory mediators released by imbalanced gut microbiota induces hormone imbalance, leading to reproductive dysfunction (96). Gut microbiota dysbiosis-induced inflammation plays a role in the development of endometriosis (127) and also disrupts the ovarian milieu, thus impairing follicular development, ovulation, and oocyte quality (128).

Furthermore, intestinal microbiota eubiosis influences the genital tract microbiota through a constant ecosystem interaction between the uterus and the vagina, which is crucial for female fertility (129, 130). It is likely that there is a gut-vagina crosstalk because microbial species, like the gram-positive Lactobacillus spp. that predominate the vaginal microbiota in physiological conditions, originate from the gut and oral probiotic administration influences vaginal microbiota composition and immunity (131). These cross-talks may be disrupted by gut dysbiosis, which may also result in uterine and vaginal dysbiosis, which may alter endometrial receptivity during implantation (98, 129). Additionally, a dysbiosis of the gut microbiota can result in the leaky-gut syndrome, which can alter the microbiota of the female genital tract (130) by causing intestinal permeability and the leakage of bacteria and bacterial products into the bloodstream (132, 133).

Overall, the gut microbiota has a multi-faceted impact on female reproduction through the modulation of hormone regulation, immune function, nutrient metabolism, inflammatory pathways, and genital tract ecosystem. Dysbiosis in the gut microbiota may disrupt these processes, potentially leading to reproductive disorders, menstrual irregularities, infertility, and other reproductive health issues.

### Gut microbiota and male reproduction

Although the field of reproductive immunology is still growing and data reporting the impact of gut microbiota on male fertility is yet accumulating, there are mounting pieces of evidence sufficiently linking gut microbiota and male reproduction. Just like in females, gut microbiota regulates male reproduction through the modulation of male sex hormones, insulin sensitivity, immune system, and testicular microbiota (**Figure 2**).

**Figure 3:**
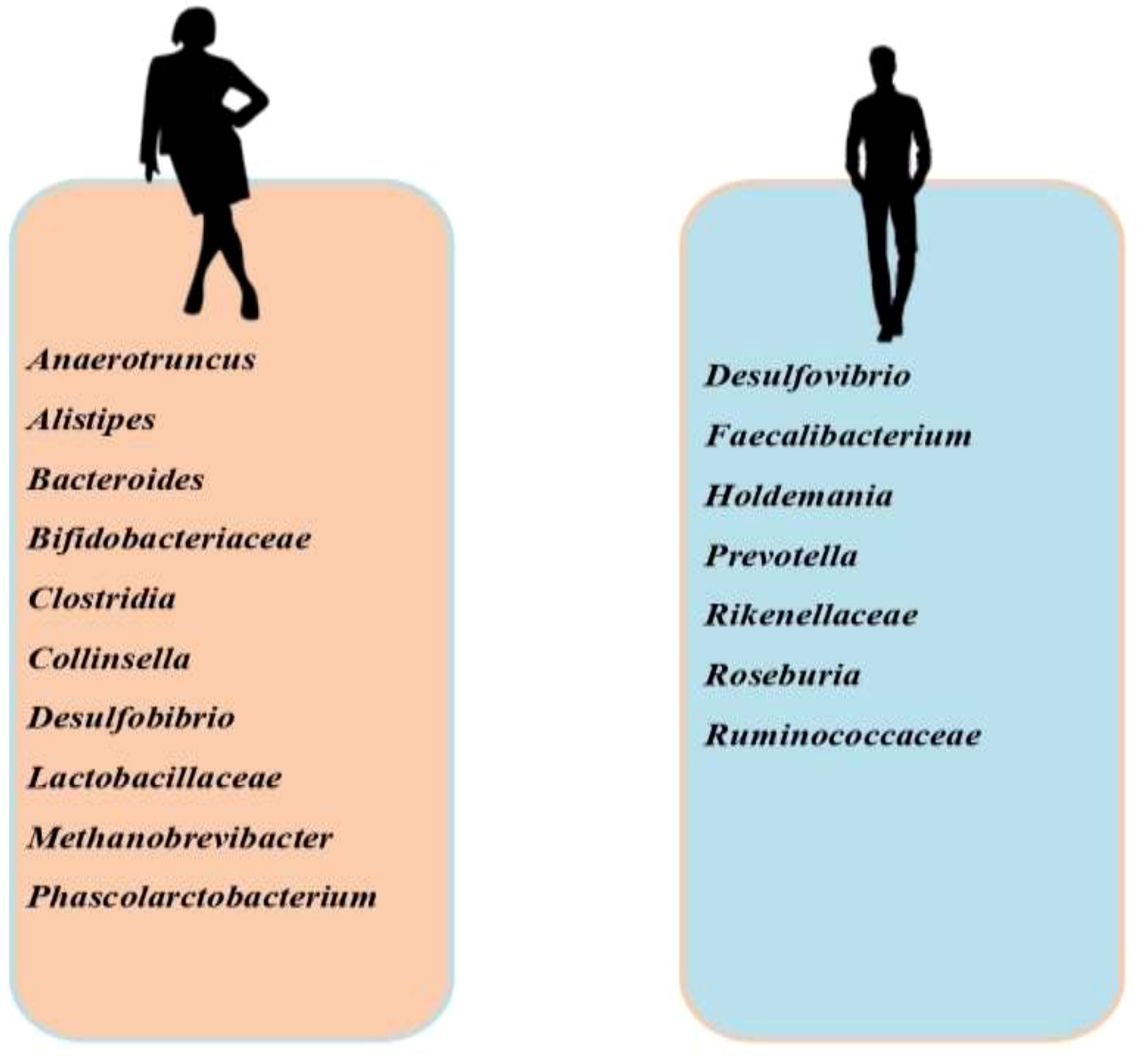
Sex-specific disparity in the prevalence of gut microbiota. Anaerotruncus, Alistipes, Bacteroides, Bifidobacteriaceae, Clostridia, Collinsella, Desulfobibrio, Lactobacillaceae, Methanobrevibacter, and Phascolarctobacterium are more abundant in females than in males, while Desulfovibrio, Faecalibacterium, Holdemania, Prevotella, Rikenellaceae, Roseburia, and Ruminococcaceae are more abundant in males than in females.

Studies have established a gender disparity with gut microbiota (**Figure 3**). *Prevotella* has been found to be more abundant in men and positively correlate with testosterone (134), while *Bacteriodes, Clostridia, Desulfobibrio* and *Methanobrevibacter* are more in women (135, 136). The link between testosterone and gut microbiota is quite complex; testosterone remodels the gut microbiota, while gut microbiota also regulates testosterone biosynthesis. In an experimental study, it was revealed that the development of blood-testis-barrier (BTB) was delayed in germ-free mice, which was accompanied by downregulation of E-cadherin, occluding, and ZO-2 in the testis (137). This impaired spermatogenesis and fertility (138). Following microbiota transplantation, there was a rise in circulating testosterone (139). These findings demonstrate the role of gut microbiota in the development of BTB, spermatogenesis, and testicular steroidogenesis. Studies have also shown that glucoronidated androgens may be excreted via the bile into the small intestine (140, 141). Gut microbiota degluconided glucoronidated testosterone and dihydrotestosterone to produce testosterone and dihydrotestosterone that are reabsorbed in the distal intestine (141). Although it remains a fact that most of the circulating testosterone is produced in the testis while a minute quantity is produced in the adrenal gland (142), *Clostridium scindens* and *Ruminococcus gnavus* have been shown to produce dihydrotestosterone and testosterone in the intestine through the conversion of glucocorticoids, Pregnenolone, and hydroxypregnenolone into androgens (143). This intestinal contribution to androgen synthesis did not alter the circulating levels of androgens. On the other hand, letrozole (aromatase inhibitor) and finasteride (5α reductase inhibitor) therapies modified gut microbiota structure and function (144, 145).

In a study by Hsu et al. (146), it was observed that there was no significant difference in the gut microbiota among patients with erectile dysfunction when compared with their counterparts without the dysfunction, however, patients with erectile dysfunction showed more abundant *Clostridium XVIII*, which has been associated with incident irritable bowel syndrome that contributes to erectile dysfunction (146). The study by Hsu et al. (146) also reported a low level of Alistipes, which has been reported to produce sulfonolipids that antagonizes von Willebrand factor receptor and block TNF-α (147) that promote endothelial injury and erectile dysfunction (148). In a study by (149), it was observed that *Allobaculum, Eubacterium, Bifidobacterium, and Anaerotruncus* were lower but TMAO, LPS, and inflammatory factors were higher in diabetic mice. This may explain the enhanced vascular inflammation, endothelial injury, and incident erectile function observed in diabetic patients.

Another mechanism through which gut microbiota modify male reproduction is the modulation of insulin sensitivity, although the current available data linking microbiota and male reproductive function are conflicting. Bäckhed et al. (150) observed a rise in body weight and development of insulin resistance 14 days post gut microbiota transplantation from the cecum to germ-free mice, however, (151) reported an improved clinical status of patients with metabolic syndrome after gut microbiota transplant. The disparity in these findings may be due to the clinical state of the subjects. It is likely that microbiota transplantation induces insulin resistance in a healthy state, but improves it in a diseased state such as metabolic syndrome. Gut microbiota disorder following LPS transfer results in low-grade inflammation (152), which induces insulin resistance via the inhibition of insulin signaling (153). It has also been shown that infertile mice with insulin resistance had a reduced level and variety of intestinal flora compared to infertile mice model without insulin resistance (152, 154, 155). It is likely that gut microbiological population predicts insulin resistance-induced testicular damage and spermatogenesis (156). Gut microbiota translocation-driven inflammation results in insulin resistance and hyperinsulinemia, which elevates lipids and suppress LH and FSH (157). Hyperinsulinemia modulates hepatic SHBG synthesis, reduces testosterone transport to the peripheral tissue, and increases circulating free testosterone, which in turn activates a negative feedback that suppresses the hypothalamic-pituitary-testicular axis and suppresses the production of LH, FSH, and testosterone (158, 159). Furthermore, microbiota dysbiosis-led insulin resistance is accompanied by leptin and ghrelin upregulation (160–162). Leptin and ghrelin impair testosterone production and modulate seminiferous tubule functions (163, 164).

Beyond the induction of an inflammatory state, gut microbiota-testes crosstalk may be mediated by the “gut leaky” hypothesis and immune activation. Ding et al. (165) demonstrated that the transplant of faecal flora from mice fed with high-fat diet to those feed with normal diet led to an increase in *Bacteriodes* and *Prevotella* in the intestine of the normal diet mice. This triggered a local inflammatory state, endotoxemia, and impairment of spermatogenesis (165). A negative correlation was also observed between sperm viability and *Bacteriodes* and *Prevotella* (165). The gut microbiota (microbial-associated molecular patterns, MAMPs, such as lipoprotein acids, lipoproteins, peptidoglycans, and lipopolysaccharide, LPS) translocates into the circulation via the hepatic portal vein or lymphatic system and gets to the testis through the testicular artery to induce hyper-immunological response, chronic inflammation, disruption of blood-testis-barrier, and testicular damage (166) by activating the innate immune cells and pattern recognition receptors-expressing epithelial cells (167) through LPS/toll like receptor 4 (TLR4), nuclear factor kappa-B (NFkB)/mitogen-activated protein kinases (MAPK), and MyD88 and TRAM-dependent signaling pathways (168). The pro-inflammatory cytokines also activate xanthine oxidase, leading to increased generation of uric acid and oxidative stress (169–171) that causes Leydig and Sertoli cells damage (172–174). In addition, accumulation of macrophages and dendritic cells in the epididymal lumen may trap normal sperm cells and trigger immunological damage (175).

The testis is not absolutely sterile as earlier thought. It contains microflora that influence its functions. Although studies on epididymal microbiota are scarce, testicular microbiota is similar to the gut microbiota, and just like the gut microbiota, it influences male reproductive functions. Testicular microbiome was observed to be reduced in diversity, especially in *Bacteroides* and *Proteobacteria*, in patients with idiopathic nonobstructive azoospermia (176). After decontamination with *Actinobacteria*, *Firmicutes*, *Blautia*, Clostridium, Bacteroidetes, and *Prevotella*, testicular studies showed a similar microbiome (177). Su et al. (178) also demonstrated a similar alteration in the testicular and gut microbiota when experimental animals were fed a high-fat diet. Zhang et al. (179) also revealed that faecal microbiota transplantation improved gut and testicular microbiota and also promoted spermatogenesis via the upregulation of glutathione peroxidase, and the protein levels of spermatogenesis-related genes in the testis (180) and arginine levels (181).

The link between gut microbiota and male reproduction has also been demonstrated in probiotic and prebiotic supplementation. Valcarce et al. (182) showed that probiotics (*Lactobacillus rhamnosus* CECT8361 and *Bifidobacterium longum* CECT7347) reduced sperm DNA fragmentation and improved sperm motility by downregulating ROS generation. These findings are similar to those observed by Abbasi et al. (183) when *Lactobacillus paracasei* B21060, oligo-fructosaccharides, arabinogalactan, and L-glutamine were administered. *Lactobacillus rhamnosus* PB01 supplementation has also been reported to improve sperm motility, normal morphology, and Leydig cells number in the testis (184). Prebiotics, such as oligofructose, have been shown to promote testosterone production and spermatogenesis (185).

Gut microbiota also modulates testicular macrophage. The testes are immune privilege organs and the macrophages are first seeded during prenatal development. Gut microbiota preserves the immune privileged testicular microenvironment by promoting anti-inflammatory cells and factors such as toll-like receptor 2 (TLR2), interleukin (IL)10 (IL-10), short chain fatty acids, dihydrotestosterone, occluding, claudins, and zona occludens; however when abnormal bacteria proliferate in large quantities, they upregulate pro-inflammatory molecules such as TLR4, TNF-α, IL-6, IL-1β, nuclear factor-kappa B (NF-kB), lipopolysaccharide (LPO), branched chain fatty acids (BCFAs), myeloid differentiation factor 88 (MyD88), and translocation associated membrane protein (TRAM) and break the immune privileged testicular microenvironment (186), hence impairing testicular functions.

## Gut microbiota and epigenetic modification

Notably, both the gut microbiota and epigenetic processes are dynamic and heavily influenced by environmental factors and diet. This suggests the possibility of shared triggers and potential link between the two in the regulation of host physiology (187). Epigenetic modifications serve as a mechanism by which mammalian cells can modulate gene expression without modifying the genetic code (188). Consequently, they play a fundamental role in enabling mammalian cells to adapt their transcriptional program in response to environmental cues. Epigenetic modifications generally associated with chromatin relaxation (euchromatin) facilitate active gene transcription, while the condensation of histone-DNA complexes (heterochromatin) indicates regions that are inaccessible and silenced (189).

Several bacteria in semen have been linked to male infertility (190). The interactions between the gastrointestinal microbiota and endocrine-disrupting compounds (EDCs) are complex and interconnected. On one hand, environmental contaminants have the potential to disrupt the composition of gastrointestinal bacteria and their metabolic activities, subsequently influencing the host’s microbial profile. On the other hand, the gastrointestinal microbiota plays a significant role in metabolizing environmental chemicals, thereby influencing their toxicity within the host. The microbiota is considered an additional organ involved in the biotransformation of xenobiotics and has an impact on the pharmacokinetics of environmental chemicals. Consequently, an altered symbiotic flora can potentially modify how chemical substances exert their toxic effects (191).

It has been shown that the intracellular pathogen Mycoplasma, which is frequently present in colonic epithelia, produces the enzymes known as DNA methyltransferases (DNMTs), which are in charge of catalyzing DNA methylation (192). Notably, these mycoplasmal DNMTs could localize within the host nucleus and induce alterations in DNA methylation at genomic regions that are typically unaffected by host-derived DNMTs. These findings suggest that microbes have the potential to directly induce unique and enduring epigenetic modifications in the host (192). Apart from *Mycoplasma*, commensal microbiota can also contribute to epigenetic modifications in the host genome through various mechanisms. One such mechanism involves the production of Short Chain Fatty Acids (SCFAs), including acetate, butyrate, and propionate. SCFAs, generated through dietary fiber digestion, play a crucial role in promoting intestinal health. Notably, butyrate acts as a major nutrient source for colonocytes and has been shown to suppress nuclear factor-B (Nf-kB) activation, thereby mitigating intestinal inflammation. Furthermore, butyrate serves as a histone deacetylase inhibitor, facilitating epigenetic remodeling in intestinal stem cells (193). It has also been demonstrated that histone deacetylase inhibition by acetate suppresses oxidative stress and NFkB-mediated inflammation to promote testicular functions viz. testosterone production and spermatogenesis (193). Acetate, a known catalyst for the removal of the acetyl group from histone to create a link between the DNA and lysine-rich histone tail, has also been shown to promote sexual function by upregulating testosterone-dependent eNOS/NO/cGMP signaling and activating Nrf2/heme oxygenase (HO)-1 via suppression of epigenetic alteration and histone modification (194).

Biotinylation, another important epigenetic process, involves the attachment of biotin to histone groups. This process plays a key role in suppressing retrotransposition and maintaining chromosomal stability. Humans rely on both dietary and bacteria-derived biotin since they are unable to synthesize it themselves. Certain commensal genera, particularly *Lactobacillus* and *Bifidobacteria*, impact the bioavailability of methyl groups through their production of folate. Folate is involved in the one-carbon metabolism cycle, regulating the availability of methyl donors and consequently affecting DNA methylation (195). Microbiota-mediated signaling through Pattern Recognition Receptors (PRRs) plays a vital role in the immunological processes occurring after birth. It is crucial for the maturation of gut-associated lymphoid tissue (GALT), the conversion of CD4(+) T cells into Foxp3(+) T-regulatory cells, and the establishment of a balanced TH1/TH2 immune response (196). Studies have shown that biotinylation enhances sperm motility (197) and the fertilization capacity of spermatozoa (198).

## Gut virome, gut microbiome, and fertility

Increasing evidence shows that gut virome is essential in shaping the composition and function of gut microbiota (199, 200). The gut viral community is dominated by prokaryotic viruses (201) such as bacteriophages that attack bacteria in a host-specific form (202). Through a phage-mediated gut microbiome modulation, gut virome alters the phenotype of the gut microbiota (203, 204). The effect of gut viruses on gut microbiota determines their impacts on fertility. Rasmussen et al. (205) demonstrated that fecal virome transfer upregulated the proliferation of *Akkermansia muciniphila*, a commensal gut, and unexpectedly enhances fertility in a mice model. It is likely that these microorganisms influence gonadal metatranscriptomics profile; however, there is a dearth of data on the gut microorganisms, bacteria or viral, that may have a significant impact on gonadal metatranscriptomics profile.

## Conclusion and future perspectives

There are existing pieces of compelling evidences, however little, which prove beyond reasonable doubts the link between the gut microbiota and reproduction. Most studies agree that gut microbiota influences gonadal functions by modulation steroid sex hormones, insulin sensitivity, immune system, and gonadal microbiota. Also, ingestion of probiotics and prebiotics also modifies gonadal functions by modulating the gut and gonadal microbiota. Although the mechanisms involved in gut microbiota-gonadal cross talk are complex and yet to be fully explored, the roles of gut microbiota, as well as probiotics and prebiotics that promote gut microbiota, should not be downplayed. Human studies validating the findings in animal models are important to curtail the reported global decline in fertility, especially for couples seeking conception. Also, it is important to investigate the gut microorganisms that may have a significant impact on gonadal metatranscriptomics profile. In addition, the role of gut virome and epididymal microbiota in reproduction should be explored.

## Author contributions

VA: Data curation, Investigation, Project administration, Resources, Writing – original draft, Writing – review & editing. BA: Data curation, Investigation, Project administration, Resources, Writing – original draft, Writing – review & editing. PA: Data curation, Investigation, Project administration, Resources, Writing – original draft, Writing – review & editing. TA: Data curation, Investigation, Project administration, Resources, Writing – original draft, Writing – review & editing. RA: Conceptualization, Data curation, Formal Analysis, Funding acquisition, Investigation, Methodology, Project administration, Resources, Software, Supervision, Validation, Visualization, Writing – original draft, Writing – review & editing.

## References

[1] Arweiler NB Netuschil L. The oral microbiota. Microbiota of the human body: implications in health and disease. (2016), 45–60. doi: 10.1007/978-3-319-31248-4_4

[2] Chen YE Fischbach MA Belkaid Y. Skin microbiota–host interactions. Nature. (2018) 553:427–36. 10.1038/nature25177 PMC6075667 29364286

[3] Chee WJ Chew SY Than LT. Vaginal microbiota and the potential of Lactobacillus derivatives in maintaining vaginal health. Microbial Cell factories. (2020) 19:203. 33160356 10.1186/s12934-020-01464-4 PMC7648308

[4] Onywera H Williamson AL Ponomarenko J Meiring TL. The penile microbiota in uncircumcised and circumcised men: relationships with HIV and human papillomavirus infections and cervicovaginal microbiota. Front Med. (2020) 7:383. doi: 10.3389/fmed.2020.00383 PMC7406686 32850898

[5] Browne HP Neville BA Forster SC Lawley TD. Transmission of the gut microbiota: spreading of health. Nat Rev Microbiol. (2017) 15:531–43. doi: 10.1038/nrmicro.2017.50 PMC5837012 28603278

[6] Sender R Fuchs S Milo R. Revised estimates for the number of human and bacteria cells in the body. PloS Biol. (2016) 14:e1002533. doi: 10.1371/journal.pbio.1002533 27541692 PMC4991899

[7] Falkow S. Who speaks for the microbes? Emerging Infect Dis. (1998) 4:495–7. doi: 10.3201/eid0403.980342 PMC2640304 9716983

[8] Koenig JE Spor A Scalfone N Fricker AD Stombaugh J Knight R. Succession of microbial consortia in the developing infant gut microbiome. Proc Natl Acad Sci United States America. (2011) 108 Suppl 1:4578–85. doi: 10.1073/pnas.1000081107 PMC3063592 20668239

[9] Yatsunenko T Rey FE Manary MJ Trehan I Dominguez-Bello MG Contreras M. Human gut microbiome viewed across age and geography. Nature. (2012) 486:222–7. doi: 10.1038/nature11053 PMC3376388 22699611

[10] Handl S Dowd SE Garcia-Mazcorro JF Steiner JM Suchodolski JS. Massive parallel 16S rRNA gene pyrosequencing reveals highly diverse fecal bacterial and fungal communities in healthy dogs and cats. FEMS Microbiol Ecol. (2011) 76:301–10. doi: 10.1111/j.1574-6941.2011.01058.x 21261668

[11] Hand D Wallis C Colyer A Penn CW. Pyrosequencing the canine faecal microbiota: breadth and depth of biodiversity. PloS One. (2013) 8:e53115. doi: 10.1371/journal.pone.0053115 23382835 PMC3561364

[12] Møller-Stray J Eriksen HM Bruheim T Kapperud G Lindstedt BA Skeie Å. Two outbreaks of diarrhoea in nurseries in Norway after farm visits, April to May 2009. Euro surveillance: Bull Europeen sur les maladies transmissibles = Eur communicable Dis Bull. (2012) 17:20321. doi: 10.2807/ese.17.47.20321-en 23231858

[13] Knetsch CW Connor TR Mutreja A van Dorp SM Sanders IM Browne HP. Whole genome sequencing reveals potential spread of Clostridium difficile between humans and farm animals in the Netherlandle genome sequencing reveals potential spread of Clostridium difficile between humans and farm animals in theto 2011. Euro surveillance: Bull Europeen sur les maladies transmissibles = Eur communicable Dis Bull. (2014) 19:20954. doi: 10.2807/1560-7917.es2014.19.45.20954 PMC4518193 25411691

[14] Toro M Retamal P Ayers S Barreto M Allard M Brown EW. Whole-Genome Sequencing Analysis of Salmonella enterica Serovar Enteritidis Isolates in Chile Provides Insights into Possible Transmission between Gulls, Poultry, and Humans. Appl Environ Microbiol. (2016) 82:6223–32. doi: 10.1128/AEM.01760-16 PMC5068155 27520817

[15] De Filippo C Cavalieri D Di Paola M Ramazzotti M Poullet JB Massart S. Impact of diet in shaping gut microbiota revealed by a comparative study in children from Europe and rural Africa. Proc Natl Acad Sci United States America. (2010) 107:14691–6. doi: 10.1073/pnas.1005963107 PMC2930426 20679230

[16] Simon O Vahjen W Scharek-Tedin L. (2003). Micro-organisms as feed additives-probiotics, in: 9th International Symposium of Digestive Physiology in Pigs, . pp. 295–318.

[17] Hill C Guarner F Reid G Gibson GR Merenstein DJ Pot B. Expert consensus document. The International Scientific Association for Probiotics and Prebiotics consensus statement on the scope and appropriate use of the term probiotic. Nat Rev Gastroenterol Hepatol. (2014) 11:506–14. doi: 10.1038/nrgastro.2014.66 24912386

[18] Deehan EC Duar RM Armet AM Perez-Muñoz ME Jin M Walter J. Modulation of the gastrointestinal microbiome with nondigestible fermentable carbohydrates to improve human health. Microbiol Spectr. (2017) 5. doi: 10.1128/microbiolspec.BAD-0019-2017 PMC11687544 28936943

[19] Schachtsiek M Hammes WP Hertel C. Characterization of Lactobacillus coryniformis DSM 20001T surface protein Cpf mediating coaggregation with and aggregation among pathogens. Appl Environ Microbiol. (2004) 70:7078–85. doi: 10.1128/AEM.70.12.7078-7085.2004 PMC535140 15574903

[20] Oelschlaeger TA. Mechanisms of probiotic actions—A review. Int J Med Microbiol. (2010) 300:57–62. doi: 10.1016/j.ijmm.2009.08.005 19783474

[21] Cremonini F Di Caro S Nista EC Bartolozzi F Capelli G Gasbarrini G. Meta-analysis: the effect of probiotic administration on antibiotic-associated diarrhoea. Alimentary Pharmacol Ther. (2002) 16:1461–7. doi: 10.1046/j.1365-2036.2002.01318.x 12182746

[22] Johnston BC Supina AL Vohra S. Probiotics for pediatric antibiotic-associated diarrhea: a meta-analysis of randomized placebo-controlled trials. CMAJ: Can Med Assoc J = J l’Association medicale Can. (2006) 175:377–83. doi: 10.1503/cmaj.051603 PMC1534112 16908901

[23] Crittenden R Payne MJ. Nutrition news. Facts and functions of prebiotics, probiotics and synbiotics. Department of Human Nutrition, K-State Research and Extension, Kansas State University; Prebiotics. In: Lee Y.K. Salminen S. Eds., Handbook of Probiotics and Prebiotics, 2nd Edition, Chap. 4., Wiley-Interscience, Hoboken, (2008). pp. 1–2, 535–82.

[24] Markowiak P Śliżewska K. Effects of probiotics, prebiotics, and synbiotics on human health. Nutrients. (2017) 9. doi: 10.3390/nu9091021 PMC5622781 28914794

[25] Natchu UC Bhatnagar S. Diarrhoea in children: identifying the cause and burden. Lancet (London England). (2013) 382:184–6. doi: 10.1016/S0140-6736(13)60941-1 23680351

[26] Koskey AM Fisher JC Eren AM Ponce-Terashima R Reis MG Blanton RE. Blautia and Prevotella sequences distinguish human and animal fecal pollution in Brazil surface waters. Environ Microbiol Rep. (2014) 6:696–704. doi: 10.1111/1758-2229.12189 25360571 PMC4247797

[27] Snelling AM Saville T Stevens D Beggs CB. Comparative evaluation of the hygienic efficacy of an ultra-rapid hand dryer vs conventional warm air hand dryers. J Appl Microbiol. (2011) 110:19–26. doi: 10.1111/j.1365-2672.2010.04838.x 20887403 PMC3017747

[28] Bull MJ Plummer NT. Part 1: the human gut microbiome in health and disease. Integr Med (Encinitas Calif.). (2014) 13:17–22. PMC4566439 26770121

[29] Dunn AB Jordan S Baker BJ Carlson NS. The maternal infant microbiome: considerations for labor and birth. MCN. Am J Maternal Child Nurs. (2017) 42:318–25. doi: 10.1097/NMC.0000000000000373 PMC5648605 28825919

[30] Nash MJ Frank DN Friedman JE. Early microbes modify immune system development and metabolic homeostasis-the “Restaurant” Hypothesis revisited. Front Endocrinol. (2017) 8:349. doi: 10.3389/fendo.2017.00349 PMC5733336 29326657

[31] McIntosh K Reed DE Schneider T Dang F Keshteli AH De Palma G. FODMAPs alter symptoms and the metabolome of patients with IBS: a randomised controlled trial. Gut. (2017) 66:1241–51. doi: 10.1136/gutjnl-2015-311339 26976734

[32] Uchida M Mogami O Matsueda K. Characteristic of milk whey culture with Propionibacterium freudenreichii ET-3 and its application to the inflammatory bowel disease therapy. Inflammopharmacology. (2007) 15:105–8. doi: 10.1007/s10787-007-1557-5 19847949

[33] Foligné B Breton J Mater D Jan G. Tracking the microbiome functionality: focus on Propionibacterium species. Gut. (2013) 62:1227–8. doi: 10.1136/gutjnl-2012-304393 23389969

[34] Etxeberria U Arias N Boqué N Macarulla MT Portillo MP Martínez JA. Reshaping faecal gut microbiota composition by the intake of trans-resveratrol and quercetin in high-fat sucrose diet-fed rats. J Nutr Biochem. (2015) 26:651–60. doi: 10.1016/j.jnutbio.2015.01.002 25762527

[35] Cheng W Lu J Li B Lin W Zhang Z Wei X. Effect of functional oligosaccharides and ordinary dietary fiber on intestinal microbiota diversity. Front Microbiol. (2017) 8:1750. doi: 10.3389/fmicb.2017.01750 28979240 PMC5611707

[36] Rodriguez-Palacios A Harding A Menghini P Himmelman C Retuerto M Nickerson KP. The artificial sweetener splenda promotes gut proteobacteria, dysbiosis, and myeloperoxidase reactivity in crohn’s disease-like ileitis. Inflammatory bowel Dis. (2018) 24:1005–20. doi: 10.1093/ibd/izy060 PMC5950546 29554272

[37] Valdes AM Walter J Segal E Spector TD. Science and Politics of Nutrition: Role of the gut microbiota in nutrition and health. BMJ. (2018) 361. doi: 10.1136/bmj.k2179 PMC6000740 29899036

[38] Zhao L Zhang F Ding X Wu G Lam YY Wang X. Gut bacteria selectively promoted by dietary fibers alleviate type 2 diabetes. Sci (New York N.Y.). (2018) 359:1151–6. doi: 10.1126/science.aao5774 29590046

[39] Chen B Chen H Shu X Yin Y Li J Qin J. Presence of segmented filamentous bacteria in human children and its potential role in the modulation of human gut immunity. Front Microbiol. (2018) 9:1403. doi: 10.3389/fmicb.2018.01403 30008704 PMC6034559

[40] Moreno-Indias I Sánchez-Alcoholado L Pérez-Martínez P Andrés-Lacueva C Cardona F Tinahones F. Red wine polyphenols modulate fecal microbiota and reduce markers of the metabolic syndrome in obese patients. Food Funct. (2016) 7:1775–87. doi: 10.1039/c5fo00886g 26599039

[41] Bonder MJ Kurilshikov A Tigchelaar EF Mujagic Z Imhann F Vila AV. The effect of host genetics on the gut microbiome. Nat Genet. (2016) 48:1407–12. doi: 10.1038/ng.3663 27694959

[42] Jackson MA Verdi S Maxan ME Shin CM Zierer J Bowyer RCE. Gut microbiota associations with common diseases and prescription medications in a population-based cohort. Nat Commun. (2018) 9:2655. doi: 10.1038/s41467-018-05184-7 29985401 PMC6037668

[43] Weersma RK Zhernakova A Fu J. Interaction between drugs and the gut microbiome. Gut. (2020) 69:1510–9. doi: 10.1136/gutjnl-2019-320204 PMC7398478 32409589

[44] Falony G Joossens M Vieira-Silva S Wang J Darzi Y Faust K. Population-level analysis of gut microbiome variation. Sci (New York N.Y.). (2016) 352:560–4. doi: 10.1126/science.aad3503 27126039

[45] Vich Vila A Collij V Sanna S Sinha T Imhann F Bourgonje AR. Impact of commonly used drugs on the composition and metabolic function of the gut microbiota. Nat Commun. (2020) 11:362. doi: 10.1038/s41467-019-14177-z 31953381 PMC6969170

[46] Imhann F Bonder MJ Vich Vila A Fu J Mujagic Z Vork L. Proton pump inhibitors affect the gut microbiome. Gut. (2016) 65:740–8. doi: 10.1136/gutjnl-2015-310376 PMC4853569 26657899

[47] Jackson MA Goodrich JK Maxan ME Freedberg DE Abrams JA Poole AC. Proton pump inhibitors alter the composition of the gut microbiota. Gut. (2016) 65:749–56. doi: 10.1136/gutjnl-2015-310861 PMC4853574 26719299

[48] Forslund K Hildebrand F Nielsen T Falony G Le Chatelier E Sunagawa S. Disentangling type 2 diabetes and metformin treatment signatures in the human gut microbiota. Nature. (2015) 528:262–6. 10.1038/nature15766 PMC4681099 26633628

[49] Wu H Esteve E Tremaroli V Khan MT Caesar R Mannerås-Holm L. Metformin alters the gut microbiome of individuals with treatment-naive type 2 diabetes, contributing to the therapeutic effects of the drug. Nat Med. (2017) 23:850–8. doi: 10.1038/nm.4345 28530702

[50] Vandeputte D Falony G Vieira-Silva S Tito RY Joossens M Raes J. Stool consistency is strongly associated with gut microbiota richness and composition, enterotypes and bacterial growth rates. Gut. (2016) 65:57–62. doi: 10.1136/gutjnl-2015-309618 26069274 PMC4717365

[51] Vandeputte D Kathagen G D’hoe K Vieira-Silva S Valles-Colomer M Sabino J. Quantitative microbiome profiling links gut community variation to microbial load. Nature. (2017) 551:507–11. doi: 10.1038/nature24460 29143816

[52] Tropini C Moss EL Merrill BD Ng KM Higginbottom SK Casavant EP. Transient osmotic perturbation causes long-term alteration to the gut microbiota. Cell. (2018) 173:1742–1754.e17. doi: 10.1016/j.cell.2018.05.008 29906449 PMC6061967

[53] Hagan T Cortese M Rouphael N Boudreau C Linde C Maddur MS. Antibiotics-driven gut microbiome perturbation alters immunity to vaccines in humans. Cell. (2019) 178:1313–1328.e13. doi: 10.1016/j.cell.2019.08.010 31491384 PMC6750738

[54] Cristofori F Dargenio VN Dargenio C Miniello VL Barone M Francavilla R. Anti-inflammatory and immunomodulatory effects of probiotics in gut inflammation: A door to the body. Front Immunol. (2021) 12:578386. doi: 10.3389/fimmu.2021.578386 33717063 PMC7953067

[55] Round JL Mazmanian SK. The gut microbiota shapes intestinal immune responses during health and disease. Nat Rev Immunol. (2009) 9:313–23. doi: 10.1038/nri2515 PMC4095778 19343057

[56] Cerf-Bensussan N Gaboriau-Routhiau V. The immune system and the gut microbiota: friends or foes? Nat Rev Immunol. (2010) 10:735–44. doi: 10.1038/nri2850 20865020

[57] Rogier EW Frantz AL Bruno ME Kaetzel CS. Secretory igA is concentrated in the outer layer of colonic mucus along with gut bacteria. Pathog (Basel Switzerland). (2014) 3:390–403. doi: 10.3390/pathogens3020390 PMC4243452 25437806

[58] Kamada N Seo SU Chen GY Núñez G. Role of the gut microbiota in immunity and inflammatory disease. Nat Rev Immunol. (2013) 13:321–35. doi: 10.1038/nri3430 23618829

[59] Formes H Bernardes JP Mann A Bayer F Pontarollo G Kiouptsi K. The gut microbiota instructs the hepatic endothelial cell transcriptome. Iscience. (2021) 24. doi: 10.1016/j.isci.2021.103092 PMC8479694 34622147

[60] Zhao S Liu W Wang J Shi J Sun Y Wang W. Akkermansia muciniphila improves metabolic profiles by reducing inflammation in chow diet-fed mice. J Mol Endocrinol. (2017) 58:1–14. doi: 10.1530/JME-16-0054 27821438

[61] Reunanen J Kainulainen V Huuskonen L Ottman N Belzer C Huhtinen H. Akkermansia muciniphila adheres to enterocytes and strengthens the integrity of the epithelial cell layer. Appl Environ Microbiol. (2015) 81:3655–62. doi: 10.1128/AEM.04050-14 PMC4421065 25795669

[62] Desai MS Seekatz AM Koropatkin NM Kamada N Hickey CA Wolter M. A dietary fiber-deprived gut microbiota degrades the colonic mucus barrier and enhances pathogen susceptibility. Cell. (2016) 167:1339–1353.e21. doi: 10.1016/j.cell.2016.10.043 27863247 PMC5131798

[63] McGuckin MA Lindén SK Sutton P Florin TH. Mucin dynamics and enteric pathogens. Nat Rev Microbiol. (2011) 9:265–78. doi: 10.1038/nrmicro2538 21407243

[64] Sonnenburg JL Xu J Leip DD Chen CH Westover BP Weatherford J. Glycan foraging in *vivo* by an intestine-adapted bacterial symbiont. Sci (New York N.Y.). (2005) 307:1955–9. doi: 10.1126/science.1109051 15790854

[65] Brownlee IA Havler ME Dettmar PW Allen A Pearson JP. Colonic mucus: secretion and turnover in relation to dietary fibre intake. Proc Nutr Soc. (2003) 62:245–9. doi: 10.1079/pns2003206 12756974

[66] Hedemann MS Theil PK Bach Knudsen KE. The thickness of the intestinal mucous layer in the colon of rats fed various sources of non-digestible carbohydrates is positively correlated with the pool of SCFA but negatively correlated with the proportion of butyric acid in digesta. Br J Nutr. (2009) 102:117–25. doi: 10.1017/S0007114508143549 19138435

[67] Earle KA Billings G Sigal M Lichtman JS Hansson GC Elias JE. Quantitative imaging of gut microbiota spatial organization. Cell Host Microbe. (2015) 18:478–88. doi: 10.1016/j.chom.2015.09.002 PMC4628835 26439864

[68] Cameron EA Sperandio V. Frenemies: signaling and nutritional integration in pathogen-microbiota-host interactions. Cell Host Microbe. (2015) 18:275–84. doi: 10.1016/j.chom.2015.08.007 PMC4567707 26355214

[69] McKenney PT Pamer EG. From hype to hope: the gut microbiota in enteric infectious disease. Cell. (2015) 163:1326–32. doi: 10.1016/j.cell.2015.11.032 PMC4672394 26638069

[70] Fu J Wei B Wen T Johansson ME Liu X Bradford E. Loss of intestinal core 1-derived O-glycans causes spontaneous colitis in mice. J Clin Invest. (2011) 121:1657–66. doi: 10.1172/JCI45538 PMC3069788 21383503

[71] Johansson ME Gustafsson JK Holmén-Larsson J Jabbar KS Xia L Xu H. Bacteria penetrate the normally impenetrable inner colon mucus layer in both murine colitis models and patients with ulcerative colitis. Gut. (2014) 63:281–91. doi: 10.1136/gutjnl-2012-303207 PMC3740207 23426893

[72] Wesemann DR Portuguese AJ Meyers RM Gallagher MP Cluff-Jones K Magee JM. Microbial colonization influences early B-lineage development in the gut lamina propria. Nature. (2013) 501:112–5. doi: 10.1038/nature12496 PMC3807868 23965619

[73] Hooper LV Littman DR Macpherson AJ. Interactions between the microbiota and the immune system. Sci (New York N.Y.). (2012) 336:1268–73. doi: 10.1126/science.1223490 PMC4420145 22674334

[74] Ueda Y Liao D Yang K Patel A Kelsoe G. T-independent activation-induced cytidine deaminase expression, class-switch recombination, and antibody production by immature/transitional 1 B cells. J Immunol (Baltimore Md.: 1950). (2007) 178:3593–601. doi: 10.4049/jimmunol.178.6.3593 PMC1955467 17339456

[75] Shaw MH Kamada N Kim YG Núñez G. Microbiota-induced IL-1β, but not IL-6, is critical for the development of steady-state TH17 cells in the intestine. J Exp Med. (2012) 209:251–8. doi: 10.1084/jem.20111703 PMC3280878 22291094

[76] Ascher S Wilms E Pontarollo G Formes H Bayer F Müller M. Gut microbiota restricts NETosis in acute mesenteric ischemia-reperfusion injury. Arteriosclerosis Thrombosis Vasc Biol. (2020) 40:2279–92. 10.1161/ATVBAHA.120.314491 PMC7484055 32611241

[77] Atarashi K Nishimura J Shima T Umesaki Y Yamamoto M Onoue M. ATP drives lamina propria T(H)17 cell differentiation. Nature. (2008) 455:808–12. doi: 10.1038/nature07240 18716618

[78] Ivanov II Atarashi K Manel N Brodie EL Shima T Karaoz U. Induction of intestinal Th17 cells by segmented filamentous bacteria. Cell. (2009) 139:485–98. doi: 10.1016/j.cell.2009.09.033 PMC2796826 19836068

[79] Atarashi K Tanoue T Shima T Imaoka A Kuwahara T Momose Y. Induction of colonic regulatory T cells by indigenous Clostridium species. Sci (New York N.Y.). (2011) 331:337–41. doi: 10.1126/science.1198469 PMC3969237 21205640

[80] Geuking MB Cahenzli J Lawson MA Ng DC Slack E Hapfelmeier S. Intestinal bacterial colonization induces mutualistic regulatory T cell responses. Immunity. (2011) 34:794–806. doi: 10.1016/j.immuni.2011.03.021 21596591

[81] Round JL Lee SM Li J Tran G Jabri B Chatila TA. The Toll-like receptor 2 pathway establishes colonization by a commensal of the human microbiota. Sci (New York N.Y.). (2011) 332:974–7. doi: 10.1126/science.1206095 PMC3164325 21512004

[82] Olszak T An D Zeissig S Vera MP Richter J Franke A. Microbial exposure during early life has persistent effects on natural killer T cell function. Sci (New York N.Y.). (2012) 336:489–93. doi: 10.1126/science.1219328 PMC3437652 22442383

[83] Hedblom GA Reiland HA Sylte MJ Johnson TJ Baumler DJ. Segmented filamentous bacteria – metabolism meets immunity. Front Microbiol. (2018) 9:1991. doi: 10.3389/fmicb.2018.01991 30197636 PMC6117376

[84] Jonsson H. Segmented filamentous bacteria in human ileostomy samples after high-fiber intake. FEMS Microbiol Lett. (2013) 342:24–9. doi: 10.1111/1574-6968.12103 23406300

[85] Yin Y Wang Y Zhu L Liu W Liao N Jiang M. Comparative analysis of the distribution of segmented filamentous bacteria in humans, mice and chickens. ISME J. (2013) 7:615–21. doi: 10.1038/ismej.2012.128 PMC3578561 23151642

[86] Mazmanian SK Round JL Kasper DL. A microbial symbiosis factor prevents intestinal inflammatory disease. Nature. (2008) 453:620–5. doi: 10.1038/nature07008 18509436

[87] O’Mahony C Scully P O’Mahony D Murphy S O’Brien F Lyons A. Commensal-induced regulatory T cells mediate protection against pathogen-stimulated NF-kappaB activation. PloS Pathog. (2008) 4:e1000112. doi: 10.1371/journal.ppat.1000112 18670628 PMC2474968

[88] Macpherson AJ Gatto D Sainsbury E Harriman GR Hengartner H Zinkernagel RM. A primitive T cell-independent mechanism of intestinal mucosal IgA responses to commensal bacteria. Sci (New York N.Y.). (2000) 288:2222–6. doi: 10.1126/science.288.5474.2222 10864873

[89] Lathrop SK Bloom SM Rao SM Nutsch K Lio CW Santacruz N. Peripheral education of the immune system by colonic commensal microbiota. Nature. (2011) 478:250–4. doi: 10.1038/nature10434 PMC3192908 21937990

[90] Hand TW Dos Santos LM Bouladoux N Molloy MJ Pagán AJ Pepper M. Acute gastrointestinal infection induces long-lived microbiota-specific T cell responses. Sci (New York N.Y.). (2012) 337:1553–6. doi: 10.1126/science.1220961 PMC3784339 22923434

[91] Slack E Hapfelmeier S Stecher B Velykoredko Y Stoel M Lawson MA. Innate and adaptive immunity cooperate flexibly to maintain host-microbiota mutualism. Sci (New York N.Y.). (2009) 325:617–20. doi: 10.1126/science.1172747 PMC3730530 19644121

[92] Baker JM Al-Nakkash L Herbst-Kralovetz MM. Estrogen-gut microbiome axis: Physiological and clinical implications. Maturitas. (2017) 103:45–53. doi: 10.1016/j.maturitas.2017.06.025 28778332

[93] Valeri F Endres K. How biological sex of the host shapes its gut microbiota. Front Neuroendocrinol. (2021) 61:100912. 33713673 10.1016/j.yfrne.2021.100912

[94] He S Li H Yu Z Zhang F Liang S Liu H. The gut microbiome and sex hormone-related diseases. Front Microbiol. (2021) 12:711137. 34650525 10.3389/fmicb.2021.711137 PMC8506209

[95] Fabozzi G Rebuzzini P Cimadomo D Allori M Franzago M Stuppia L. Endocrine-disrupting chemicals, gut microbiota, and human (In) fertility—It is time to consider the triad. Cells. (2022) 11:p.3335. 10.3390/cells11213335 PMC9654651 36359730

[96] Qi X Yun C Pang Y Qiao J. The impact of the gut microbiota on the reproductive and metabolic endocrine system. Gut Microbes. (2021) 13:1894070. 33722164 10.1080/19490976.2021.1894070 PMC7971312

[97] Fu X Han H Li Y Xu B Dai W Zhang Y. Di-(2-ethylhexyl) phthalate exposure induces female reproductive toxicity and alters the intestinal microbiota community structure and fecal metabolite profile in mice. Environ Toxicol. (2021) 36:1226–42. doi: 10.1002/tox.23121 PMC8251547 33665894

[98] Wang J Li Z Ma X Du L Jia Z Cui X. Translocation of vaginal microbiota is involved in impairment and protection of uterine health. Nat Commun. (2021) 12:4191. 34234149 10.1038/s41467-021-24516-8 PMC8263591

[99] Plottel CS Blaser MJ. Microbiome and Malignancy. Cell Host Microbe. (2011) 10:324–35. 10.1016/j.chom.2011.10.003 PMC3264051 22018233

[100] Ervin SM Li H Lim L Roberts LR Liang X Mani S. Gut microbial beta-glucuronidases reactivate estrogens as components of the estrobolome that reactivate estrogens. J Biol Chem. (2019) 294:18586–99. 10.1074/jbc.RA119.010950 PMC6901331 31636122

[101] Maffei S Forini F Canale P Nicolini G Guiducci L. Gut microbiota and sex hormones: crosstalking players in cardiometabolic and cardiovascular disease. Int J Mol Sci. (2022) 23:7154. 35806159 10.3390/ijms23137154 PMC9266921

[102] Garcia-Penarrubia P Ruiz-Alcaraz AJ Martinez-Esparza M Marin P MaChado-Linde F. Hypothetical roadmap towards endometriosis: Prenatal endocrine-disrupting chemical pollutant exposure, anogenital distance, gut-genital microbiota and subclinical infections. Hum Reprod Update. (2020) 26:214–46. doi: 10.1093/humupd/dmz044 32108227

[103] Jiang I Yong PJ Allaire C Bedaiwy MA. Intricate connections between the microbiota and endometriosis. Int J Mol Sci. (2021) 22:5644. 34073257 10.3390/ijms22115644 PMC8198999

[104] Saad MJ Santos A Prada PO. Linking gut microbiota and inflammation to obesity and insulin resistance. Physiology. (2016) 31:283–93. 10.1152/physiol.00041.2015 27252163

[105] Guo J Shao J Yang Y Niu X Liao J Zhao Q. Gut microbiota in patients with polycystic ovary syndrome: A systematic review. Reprod Sci. (2022) 29:69–83. doi: 10.1007/s43032-020-00430-0 33409871

[106] Poppe K. Management of Endocrine Disease: Thyroid and female infertility: More questions than answers? Eur J Endocrinol. (2021) 184:R123–35. doi: 10.1530/EJE-20-1284 33460394

[107] Twig G Shina A Amital H Shoenfeld Y. Pathogenesis of infertility and recurrent pregnancy loss in thyroid autoimmunity. J Autoimmun. (2012) 38:J275–81. doi: 10.1016/j.jaut.2011.11.014 22218218

[108] Wang JW Liao XX Li T. Thyroid autoimmunity in adverse fertility and pregnancy outcomes: timing of assisted reproductive technology in AITD women. J Transl Int Med. (2021) 9:76–83. doi: 10.2478/jtim-2021-0001 34497747 PMC8386333

[109] Rooks MG Garrett WS. Gut microbiota, metabolites and host immunity. Nat Rev Immunol. (2016) 16:341–52. 10.1038/nri.2016.42 PMC5541232 27231050

[110] Qi X Yun C Sun L Xia J Wu Q Wang Y. Publisher Correction: Gut microbiota-bile acid-interleukin-22 axis orchestrates polycystic ovary syndrome. Nat Med. (2019) 25:1459. doi: 10.1038/s41591-019-0562-8 31471570

[111] Kriebs A. IL-22 links gut microbiota to PCOS. Nat Rev Endocrinol. (2019) 15:565. 31431732 10.1038/s41574-019-0255-x

[112] He FF Li YM. Role of gut microbiota in the development of insulin resistance and the mechanism underlying polycystic ovary syndrome: A review. J Ovarian Res. (2020) 13:73. doi: 10.1186/s13048-020-00670-3 32552864 PMC7301991

[113] Scheithauer TPM Rampanelli E Nieuwdorp M Vallance BA Verchere CB van Raalte DH. Gut microbiota as a trigger for metabolic inflammation in obesity and type 2 diabetes. Front Immunol. (2020) 11:571731. 33178196 10.3389/fimmu.2020.571731 PMC7596417

[114] Yurtdas G Akdevelioglu Y. A new approach to polycystic ovary syndrome: the gut microbiota. J Am Coll Nutr. (2020) 39:371–82. 10.1080/07315724.2019.1657515 31513473

[115] Giampaolino P Foreste V Di Filippo C Gallo A Mercorio A Serafino P. Microbiome and PCOS: state-of-art and future aspects. Int J Mol Sci. (2021) 22:2048. 33669557 10.3390/ijms22042048 PMC7922491

[116] de Rivero Vaccari JP. The inflammasome in reproductive biology: A promising target for novel therapies. Front Endocrinol. (2020) 11:8. 10.3389/fendo.2020.00008 PMC6997205 32047476

[117] Afolabi OA Hamed MA Anyogu DC Adeyemi DH Odetayo AF Akhigbe RE. Atorvastatin-mediated downregulation of VCAM-1 and XO/UA/caspase 3 signaling averts oxidative damage and apoptosis induced by ovarian ischaemia/reperfusion injury. Redox Rep. (2022) 27:212–20. doi: 10.1080/13510002.2022.2129192 PMC9553180 36200598

[118] Wu J Zhuo Y Liu Y Chen Y Ning Y Yao J. Association between premature ovarian insufficiency and gut microbiota. BMC Pregnancy Childbirth. (2021) 21:418. 34090383 10.1186/s12884-021-03855-w PMC8180047

[119] Jiang L Fei H Tong J Zhou J Zhu J Jin X. Hormone replacement therapy reverses gut microbiome and serum metabolome alterations in premature ovarian insufficiency. Front Endocrinol. (2021) 12:794496. 10.3389/fendo.2021.794496 PMC8733385 35002971

[120] Abrams ET Miller EM. The roles of the immune system in Women’s reproduction: Evolutionary constraints and life history trade-offs. Am J Phys anthropology. (2011) 146:134–54. 10.1002/ajpa.21621 22101690

[121] Martinez-Guryn K Hubert N Frazier K Urlass S Musch MW Ojeda P. Small intestine microbiota regulate host digestive and absorptive adaptive responses to dietary lipids. Cell Host Microbe. (2018) 23:pp.458–469. doi: 10.1016/j.chom.2018.03.011 PMC5912695 29649441

[122] Bhowmik D Chiranjib K Kumar S. A potential medicinal importance of zinc in human health and chronic. Int J Pharm. (2010) 1:05–11.

[123] Hostetler CE Kincaid RL Mirando MA. The role of essential trace elements in embryonic and fetal development in livestock. Veterinary J. (2003) 166:125–39. 10.1016/s1090-0233(02)00310-6 12902178

[124] Kumar S Pandey AK AbdulRazzaque WA Dwivedi DK. Importance of micro minerals in reproductive performance of livestock. Veterinary World. (2011) 4:230.

[125] Mancini A Bruno C Vergani E d’Abate C Giacchi E Silvestrini A. Oxidative stress and low-grade inflammation in polycystic ovary syndrome: controversies and new insights. Int J Mol Sci. (2021) 22:p.1667. doi: 10.3390/ijms22041667 PMC7915804 33562271

[126] Fabozzi G Verdone G Allori M Cimadomo D Tatone C Stuppia L. Personalized nutrition in the management of female infertility: new insights on chronic low-grade inflammation. Nutrients. (2022) 14:1918. doi: 10.3390/nu14091918 35565885 PMC9105997

[127] Laux-Biehlmann A d’Hooghe T Zollner TM. Menstruation pulls the trigger for inflammation and pain in endometriosis. Trends Pharmacol Sci. (2015) 36:270–6. 10.1016/j.tips.2015.03.004 25899467

[128] Weiss G Goldsmith LT Taylor RN Bellet D Taylor HS. Inflammation in reproductive disorders. Reprod Sci. (2009) 16:216–29. 10.1177/1933719108330087 PMC3107847 19208790

[129] Benner M Ferwerda G Joosten I van der Molen RG. How uterine microbiota might be responsible for a receptive, fertile endometrium. Hum Reprod Update. (2018) 24:393–415. 29668899 10.1093/humupd/dmy012

[130] Amabebe E Anumba DOC. Female gut and genital tract microbiota-induced crosstalk and differential effects of short-chain fatty acids on immune sequelae. Front Immunol. (2020) 11:2184. 33013918 10.3389/fimmu.2020.02184 PMC7511578

[131] Amabebe E Anumba DOC. The vaginal microenvironment: the physiologic role of lactobacilli. Front Med. (2018) 5:181. 10.3389/fmed.2018.00181 PMC6008313 29951482

[132] Afolabi AO Akhigbe TM Odetayo AF Anyogu DC Hamed MA Akhigbe RE. Restoration of hepatic and intestinal integrity by Phyllanthus amarus is dependent on Bax/caspase 3 modulation in intestinal ischemia-/reperfusion-induced injury. Molecules. (2022) 27:5073. doi: 10.3390/molecules27165073 36014309 PMC9413108

[133] Afolabi OA Akhigbe TM Akhigbe RE Alabi BA Gbolagun OT Taiwo ME. Methanolic Moringa oleifera leaf extract protects against epithelial barrier damage and enteric bacterial translocation in intestinal I/R: Possible role of caspase 3. Front Pharmacol. (2022) 13:989023. doi: 10.3389/fphar.2022.989023 36210817 PMC9546449

[134] Haro C Rangel-Zúñiga OA Alcalá-Díaz JF Gómez-Delgado F Pérez-Martínez P Delgado-Lista J. Intestinal microbiota is influenced by gender and body mass index. PloS One. (2016) 11(5):e0154090. doi: 10.1371/journal.pone.0154090 27228093 PMC4881937

[135] Gloux K Berteau O El Oumami H Béguet F Leclerc M Doré J. A metagenomic β-glucuronidase uncovers a core adaptive function of the human intestinal microbiome. Proc Natl Acad Sci USA. (2011) 108:4539–46. 10.1073/pnas.1000066107 PMC3063586 20615998

[136] Choi S Hwang YJ Shin MJ Yi H. Difference in the gut microbiome between ovariectomy-induced obesity and diet-induced obesity. J Microbiol Biotechnol. (2017) 27:2228–36. 10.4014/jmb.1710.10001 29121700

[137] Al-Asmakh M Stukenborg JB Reda A Anuar F Strand ML Hedin L. The gut microbiota and developmental programming of the testis in mice. PloS One. (2014) 9(8):e103809. doi: 10.1371/journal.pone.0103809 25118984 PMC4132106

[138] Griswold MD. Interactions between germ cells and Sertoli cells in the testis. Biol Reprod. (1995) 52:211–6. 10.1095/biolreprod52.2.211 7711190

[139] Markle JG Frank DN Mortin-Toth S Robertson CE Feazel LM Rolle-Kampczyk U. Sex differences in the gut microbiome drive hormone-dependent regulation of autoimmunity. Science. (2013) 339(6123):1084–8. 10.1126/science.1233521 23328391

[140] Bélanger A Pelletier G Labrie F Barbier O Chouinard S. Inactivation of androgens by UDP-glucuronosyltransferase enzymes in humans. Trends Endocrinol Metab. (2003) 14:473–9. 10.1016/j.tem.2003.10.005 14643063

[141] Colldén H Landin A Wallenius V. The gut microbiota is a major regulator of androgen metabolism in intestinal contents. Am J Physiol Endocrinol Metab. (2019) 317:E1182–92. doi: 10.1152/ajpendo.00338.2019 PMC6962501 31689143

[142] Ajayi AF Akhigbe RE. The physiology of male reproduction: Impact of drugs and their abuse on male fertility. Andrologia. (2020) 00:e13672. doi: 10.1111/and.13672 32542870

[143] Ridlon JM Ikegawa S Alves JM Zhou B Kobayashi A Iida T. Clostridium scindens: a human gut microbe with a high potential to convert glucocorticoids into androgens. J Lipid Res. (2013) 54(9):2437–49. doi: 10.1194/jlr.M038869 PMC3735941 23772041

[144] Arroyo P Ho BS Sau L Kelley ST Thackray VG. Letrozole treatment of pubertal female mice results in activational effects on reproduction, metabolism and the gut microbiome. PloS One. (2019) 14:e0223274. doi: 10.1371/journal.pone.0223274 31568518 PMC6768472

[145] Diviccaro S Giatti S Borgo F Barcella M Borghi E Trejo JL. Treatment of male rats with finasteride, an inhibitor of 5alpha-reductase enzyme, induces long-lasting effects on depressive-like behavior, hippocampal neurogenesis, neuroinflammation and gut microbiota composition. Psychoneuroendocrinology. (2019) 99:206–15. doi: 10.1016/j.psyneuen.2018.09.021 30265917

[146] Hsu CY Lin CL Kao CH. Irritable bowel syndrome is associated not only with organic but also psychogenic erectile dysfunction. Int J Impot Res. (2015) 27:233–8. 10.1038/ijir.2015.25 26548409

[147] Maeda J Nishida M Takikawa H Yoshida H Azuma T Yoshida M. Inhibitory effects of sulfobacin B on DNA polymerase and inflammation. Int J Mol Med. (2010) 26(5):751–758. 20878098 10.3892/ijmm_00000522

[148] Okamoto T Hatakeyama S Imai A. The association between gut microbiome and erectile dysfunction: a community-based crosssectional study in Japan. Int Urol Nephrol. (2020) 52:1421–8. 10.1007/s11255-020-02443-9 32193686

[149] Li H Qi T Huang ZS. Relationship between gut microbiota and type 2 diabetic erectile dysfunction in Sprague-Dawley rats. J Huazhong Univ Sci Technolog Med Sci. (2017) 37:523–30. 10.1007/s11596-017-1767-z 28786059

[150] Bäckhed F Ding H Wang T. The gut microbiota as an environmental factor that regulates fat storage. Proc Natl Acad Sci USA. (2004) 101:15718–23. 10.1073/pnas.0407076101 PMC524219 15505215

[151] Vrieze A Van Nood E Holleman F. Transfer of intestinal microbiota from lean donors increases insulin sensitivity in individuals with metabolic syndrome. Gastroenterology. (2012) 143:913–916.e917. doi: 10.1053/j.gastro.2012.06.031 22728514

[152] Pedersen HK Gudmundsdottir V Nielsen HB. Human gut microbes impact host serum metabolome and insulin sensitivity. Nature. (2016) 535:376–81. 10.1038/nature18646 27409811

[153] Hawkesworth S Moore SE Fulford AJ. Evidence for metabolic endotoxemia in obese and diabetic Gambian women. Nutr Diabetes. (2013) 3:e83. doi: 10.1038/nutd.2013.24 23978817 PMC3759130

[154] Andreasen AS Larsen N Pedersen-Skovsgaard T Berg RM Møller K Svendsen KD. Effects of Lactobacillus acidophilus NCFM on insulin sensitivity and the systemic inflammatory response in human subjects. Br J Nutr. (2010) 104(12):1831–8. 10.1017/S0007114510002874 20815975

[155] Lang UE Beglinger C Schweinfurth N Walter M Borgwardt S. Nutritional aspects of depression. Cell Physiol Biochem. (2015) 37:1029–1043. 26402520 10.1159/000430229

[156] Zhu Y Du Q Jiao N. Catalpol ameliorates diabetes-induced testicular injury and modulates gut microbiota. Life Sci. (2021) 267:118881. 33310037 10.1016/j.lfs.2020.118881

[157] Chosich J Bradford AP Allshouse AA Reusch JE Santoro N Schauer IE. Acute recapitulation of the hyperinsulinemia and hyperlipidemia characteristic of metabolic syndrome suppresses gonadotropins. Obesity. (2017) 25:553–56. 10.1002/oby.21754 PMC5323271 28158916

[158] Pitteloud N Hardin M Dwyer AA Valassi E Yialamas M Elahi D. Increasing insulin resistance is associated with a decrease in Leydig cell testosterone secretion in men. J Clin Endocrinol Metab. (2005) 90(5):2636–41. 10.1210/jc.2004-2190 15713702

[159] Hawksworth DJ Burnett AL. Nonalcoholic fatty liver disease, male sexual dysfunction, and infertility: common links, common problems. Sex Med Rev. (2020) 8:274–85. doi: 10.1016/j.sxmr.2019.01.002 30898592

[160] Sahin-Efe A Katsikeris F Mantzoros CS. Advances in adipokines. Metabolism. (2012) 61:1659–65. 10.1016/j.metabol.2012.09.001 23021039

[161] Lin T Li S Xu H Zhou H Feng R Liu W. Gastrointestinal hormone secretion in women with polycystic ovary syndrome: an observational study. Hum Reprod. (2015) 30(11):2639–44. doi: 10.1093/humrep/dev231 26373789

[162] Torres PJ Siakowska M Banaszewska B Pawelczyk L Duleba AJ Kelley ST. Gut microbial diversity in women with polycystic ovary syndrome correlates with hyperandrogenism. J Clin Endocrinol Metab. (2018) 103(4):1502–11. 10.1210/jc.2017-02153 PMC6276580 29370410

[163] Barreiro ML Tena-Sempere M. Ghrelin and reproduction: a novel signal linking energy status and fertility? Mol Cell Endocrinol. (2004) 226:1–9. 15488999 10.1016/j.mce.2004.07.015

[164] Moreira BP Monteiro MP Sousa M Oliveira PF Alves MG. Insights into leptin signaling and male reproductive health: the missing link between overweight and subfertility? Biochem J. (2018) 475:3535–3560. doi: 10.1042/BCJ20180631 30459203

[165] Ding N Zhang X Di Zhang X Jing J Liu SS Mu YP. Impairment of spermatogenesis and sperm motility by the high-fat diet-induced dysbiosis of gut microbes. Gut. (2020) 69(9):1608–19. doi: 10.1136/gutjnl-2019-319127 PMC7456731 31900292

[166] Kaur D Patiyal S Sharma N Usmani SS Raghava GPS. PRRDB 2.0: a comprehensive database of pattern-recognition receptors and their ligands. Database J Biol Database Curation. (2019) 2019:baz076. doi: 10.1093/database/baz076 PMC6597477 31250014

[167] Zhao S Zhu W Xue S Han D. Testicular defense systems: immune privilege and innate immunity. Cell Mol Immunol. (2014) 11:428–37. 10.1038/cmi.2014.38 PMC4197207 24954222

[168] Sonnex C. Toll-like receptors and genital tract infection. Int J STD AIDS. (2010) 21:153–7. 10.1258/ijsa.2009.009525 20215617

[169] Hamed MA Aremu AO Akhigbe RE. Concomitant administration of HAART aggravates anti-Koch-induced oxidative hepatorenal damage via dysregulation of glutathione and elevation of uric acid production. Biomedicine Pharmacotherapy. (2021) 137:111309. doi: 10.1016/j.biopha.2021.111309 33524784

[170] Akhigbe RE Ajayi LO Ajayi AF. Codeine exerts cardiorenal injury via upregulation of adenine deaminase/xanthine oxidase and caspase 3 signaling. Life Sci. (2021) 273:118717. 33159958 10.1016/j.lfs.2020.118717

[171] Hamed MA Akhigbe RE Aremu AO Odetayo AF. Zinc normalizes hepatic lipid handling via modulation of ADA/XO/UA pathway and caspase 3 signaling in highly active antiretroviral therapy-treated Wistar rats. Chemico-Biological Interact. (2022) 368:110233. doi: 10.1016/j.cbi.2022.110233 36309141

[172] Sarkar O Bahrainwala J Chandrasekaran S Kothari S Mathur PP Agarwal A. Impact of inflammation on male fertility. Front Biosci Elite Ed. (2011) 3:89–95. 21196288 10.2741/e223

[173] Saka WA Akhigbe RE Abidoye AO Dare OS Adekunle AO. Suppression of uric acid generation and blockade of glutathione dysregulation by L-arginine ameliorates dichlorvos-induced oxidative hepatorenal damage in rats. Biomedicine Pharmacotherapy. (2021) 138:111443. doi: 10.1016/j.biopha.2021.111443 33667786

[174] Akhigbe RE Hamed MA Aremu AO. HAART exacerbates testicular damage and impaired spermatogenesis in anti-Koch-treated rats via dysregulation of lactate transport and glutathione content. Reprod Toxicol. (2021) 103:96–107. doi: 10.1016/j.reprotox.2021.06.007 34118364

[175] Zheng W Zhang S Chen X Jiang S Li Z Li M. Case report: dendritic cells and macrophages capture sperm in chronically inflamed human epididymis. Front Immunol. (2021) 12:629680. 33708220 10.3389/fimmu.2021.629680 PMC7942197

[176] Alfano M Ferrarese R Locatelli I Ventimiglia E Ippolito S Gallina P. Testicular microbiome in azoospermic men-first evidence of the impact of an altered microenvironment. Hum Reprod. (2018) 33:1212–7. 10.1093/humrep/dey116 PMC6012977 29850857

[177] Molina NM Plaza-Díaz J Vilchez-Vargas R Sola-Leyva A Vargas E Mendoza-Tesarik R. Assessing the testicular sperm microbiome: a low-biomass site with abundant contamination. Reprod BioMed Online. (2021) 43:523–31. 10.1016/j.rbmo.2021.06.021 34344601

[178] Su Y He L Hu Z Li Y Zhang Y Fan Z. Obesity causes abrupt changes in the testicular microbiota and sperm motility of zebrafish. Front Immunol. (2021) 12:639239. 34248933 10.3389/fimmu.2021.639239 PMC8268156

[179] Zhang C Xiong B Chen L Ge W Yin S Feng Y. Rescue of male fertility following faecal microbiota transplantation from alginate oligosaccharidedosed mice. Gut. (2020) 70:2213–5. 10.1136/gutjnl-2020-323593 PMC8515102 33443023

[180] Zhang P Feng Y Li L Ge W Yu S Hao Y. Improvement in sperm quality and spermatogenesis following faecal microbiota transplantat oligosaccharide dosed mice. Gut. (2021) 70:222–5. 10.1136/gutjnl-2020-320992 PMC7788261 32303608

[181] Zhao Q Huang JF Cheng Y Dai MY Zhu WF Yang XW. Polyamine metabolism links gut microbiota and testicular dysfunction. Microbiome. (2021) 9(1):224. 34758869 10.1186/s40168-021-01157-z PMC8582214

[182] Valcarce DG Genovés S Riesco MF Martorell P Herráez MP Ramón D. Probiotic administration improves sperm quality in asthenozoospermic human donors. Benef Microbes. (2017) 8(2):193–206. 28343402 10.3920/BM2016.0122

[183] Abbasi B Abbasi H Niroumand H. Synbiotic (FamiLact) administration in idiopathic male infertility enhances sperm quality, DNA integrity, and chromatin status: a triple-blinded randomized clinical trial. Int J Reprod Biomed. (2021) 19:235–44. doi: 10.18502/ijrm.v19i3.8571 PMC8023005 33842820

[184] Dardmeh F Alipour H Gazerani P van der Horst G Brandsborg E Nielsen HI. Lactobacillus rhamnosus PB01 (DSM 14870) supplementation affects markers of sperm kinematic parameters in a diet-induced obesity mice model. PloS One. (2017) 12:e0185964. doi: 10.1371/journal.pone.0185964 29016685 PMC5634625

[185] Rodrigues LE Kishibe MM Keller R. Prebiotics mannanoligosaccharides accelerate sexual maturity in rats: a randomize preclinical study. Vet World. (2021) 14:1210–9. 10.14202/vetworld.2021.1210-1219 PMC8243662 34220123

[186] Cai H Cao X Qin D Liu Y Liu Y Hua J. Gut microbiota supports male reproduction via nutrition, immunity, and signaling. Front Microbiol. (2022) 13:977574. 36060736 10.3389/fmicb.2022.977574 PMC9434149

[187] Mehta V Nagu P Inbaraj BS Sharma M Parashar A Sridhar K. Epigenetics and gut microbiota crosstalk: A potential factor in pathogenesis of cardiovascular disorders. Bioengineering. (2022) 9:12. doi: 10.3390/bioengineering9120798 36551003 PMC9774431

[188] Akhigbe R Ajayi A. The impact of reactive oxygen species in the development of cardiometabolic disorders: a review. Lipids Health Dis. (2021) 20:23. 33639960 10.1186/s12944-021-01435-7 PMC7916299

[189] Woo V Alenghat T. Epigenetic regulation by gut microbiota. Gut Microbes. (2022) 14:2022407. doi: 10.1080/19490976.2021.2022407 35000562 PMC8744890

[190] Weng S-L Chiu C-M Lin F-M Huang W-C Liang C Yang T. Bacterial communities in semen from men of infertile couples: metagenomic sequencing reveals relationships of seminal microbiota to semen quality. PloS One. (2014) 9:e110152. doi: 10.1371/journal.pone.0110152 25340531 PMC4207690

[191] Fabozzi G Rebuzzini P Cimadomo D Allori M Franzago M Stuppia L. Endocrine-disrupting chemicals, gut microbiota, and human (In)Fertility—It is time to consider the triad. Cells. (2022) 21. doi: 10.3390/cells11213335 PMC9654651 36359730

[192] Bierne H Hamon M Cossart P. Epigenetics and bacterial infections. Cold Spring Harbor Perspect Med. (2012) 2:a010272. doi: 10.1101/cshperspect.a010272 PMC3543073 23209181

[193] Besong EE Akhigbe TM Obimma JN Obembe OO Akhigbe RE. Acetate abates arsenic-induced male reproductive toxicity by suppressing HDAC and uric acid–driven oxido-inflammatory NF k B/iNOS/NO response in rats. Biol Trace Element Res. (2023) 19:1–6. 10.1007/s12011-023-03860-4 37726447

[194] Besong EE Ashonibare PJ Akhigbe TM Obimma JN Akhigbe RE. Sodium acetate abates lead-induced sexual dysfunction by upregulating testosterone-dependent eNOS/NO/cGMP signaling and activating Nrf2/HO-1 in male Wistar rat. Naunyn-Schmiedeberg’s Arch Pharmacol. (2023) 1:1–1. 10.1007/s00210-023-02696-y 37658211

[195] Lopez-Siles M Martinez-Medina M Busquets D Sabat-Mir M Duncan SH Flint HJ. Mucosa-associated Faecalibacterium prausnitzii and Escherichia coli co-abundance can distinguish Irritable Bowel Syndrome and Inflammatory Bowel Disease phenotypes. Int J Med Microbiology: IJMM. (2014) 304:464–75. 10.1016/j.ijmm.2014.02.009 24713205

[196] Fofanova TY Petrosino JF Kellermayer R. Microbiome–epigenome interactions and the environmental origins of inflammatory bowel diseases. J Pediatr Gastroenterol Nutr. (2016) 62:208. 26308318 10.1097/MPG.0000000000000950 PMC4724338

[197] Kalthur G Salian SR Keyvanifard F Sreedharan S Thomas JS Kumar P. Supplementation of biotin to sperm preparation medium increases the motility and longevity in cryopreserved human spermatozoa. J assisted Reprod Genet. (2012) 29:631–5. 10.1007/s10815-012-9760-8 PMC3401256 22527895

[198] Salian SR Nayak G Kumari S Patel S Gowda S Shenoy Y. Supplementation of biotin to sperm preparation medium enhances fertilizing ability of spermatozoa and improves preimplantation embryo development. J Assisted Reprod Genet. (2019) 36:255–66. 10.1007/s10815-018-1323-1 PMC6420554 30284103

[199] Scarpellini E Ianiro G Attili F Bassanelli C De Santis A Gasbarrini A. The human gut microbiota and virome: potential therapeutic implications. Digestive Liver Dis. (2015) 47:1007–12. 10.1016/j.dld.2015.07.008 PMC7185617 26257129

[200] Howe A Ringus DL Williams RJ Choo Z-N Greenwald SM Owens SM. Divergent responses of viral and bacterial communities in the gut microbiome to dietary disturbances in mice. Isme J. (2016) 10:1217–27. 10.1038/ismej.2015.183 PMC5029215 26473721

[201] Reyes A Haynes M Hanson N Angly FE Heath AC Rohwer F. Viruses in the faecal microbiota of monozygotic twins and their mothers. Nature. (2010) 466:334–8. 10.1038/nature09199 PMC2919852 20631792

[202] Barrangou R Yoon S-S Breidt F Fleming HP Klaenhammer TR. Characterization of six Leuconostoc fallax bacteriophages isolated from an industrial sauerkraut fermentation. Appl Environ Microbiol. (2002) 68:5452–8. 10.1128/AEM.68.11.5452-5458.2002 PMC129880 12406737

[203] Stone E Campbell K Grant I McAuliffe O. Understanding and exploiting phage–host interactions. Viruses. (2019) 11:1–26. 10.3390/v11060567 PMC6630733 31216787

[204] Rasmussen TS Koefoed AK Jakobsen RR Deng L Castro-Mejía JL Brunse A. Bacteriophage-mediated manipulation of the gut microbiome – promises and presents limitations. FEMS Microbiol Rev. (2020) 44:507–21. 10.1093/femsre/fuaa020 32495834

[205] Rasmussen TS Mentzel CM Danielsen MR Jakobsen RR Zachariassen LS Castro Mejia JL. Fecal virome transfer improves proliferation of commensal gut Akkermansia muciniphila and unexpectedly enhances the fertility rate in laboratory mice. Gut Microbes. (2023) 15:2208504. doi: 10.1080/19490976.2023.2208504 37150906 PMC10167882

